# Reaching the “Hard-to-Reach” Sexual and Gender Diverse Communities for Population-Based Research in Cancer Prevention and Control: Methods for Online Survey Data Collection and Management

**DOI:** 10.3389/fonc.2022.841951

**Published:** 2022-06-08

**Authors:** Katie J. Myers, Talya Jaffe, Deborah A. Kanda, V. Shane Pankratz, Bernard Tawfik, Emily Wu, Molly E. McClain, Shiraz I. Mishra, Miria Kano, Purnima Madhivanan, Prajakta Adsul

**Affiliations:** ^1^ Comprehensive Cancer Center, University of New Mexico, Albuquerque, NM, United States; ^2^ Department of Internal Medicine, School of Medicine, University of New Mexico, Albuquerque, NM, United States; ^3^ Department of Obstetrics and Gynecology, School of Medicine, University of New Mexico, Albuquerque, NM, United States; ^4^ Department of Family and Community Medicine, School of Medicine, University of New Mexico, Albuquerque, NM, United States; ^5^ Department of Pediatrics, School of Medicine, University of New Mexico, Albuquerque, NM, United States; ^6^ Department of Health Promotion Sciences, Mel & Enid Zuckerman College of Public Health University of Arizona, Tucson, AZ, United States; ^7^ Comprehensive Cancer Center, University of Arizona, Tucson, AZ, United States; ^8^ Public Health Research Institute of India, Mysuru, India

**Keywords:** cancer screening (MeSH), sexual orientation, gender identity (MeSH), recruitment, cancer, cancer prevention

## Abstract

**Purpose:**

Around 5% of United States (U.S.) population identifies as Sexual and Gender Diverse (SGD), yet there is limited research around cancer prevention among these populations. We present multi-pronged, low-cost, and systematic recruitment strategies used to reach SGD communities in New Mexico (NM), a state that is both largely rural and racially/ethnically classified as a “majority-minority” state.

**Methods:**

Our recruitment focused on using: (1) Every Door Direct Mail (EDDM) program, by the United States Postal Services (USPS); (2) Google and Facebook advertisements; (3) Organizational outreach *via* emails to publicly available SGD-friendly business contacts; (4) Personal outreach *via* flyers at clinical and community settings across NM. Guided by previous research, we provide detailed descriptions on using strategies to check for fraudulent and suspicious online responses, that ensure data integrity.

**Results:**

A total of 27,369 flyers were distributed through the EDDM program and 436,177 impressions were made through the Google and Facebook ads. We received a total of 6,920 responses on the eligibility survey. For the 5,037 eligible respondents, we received 3,120 (61.9%) complete responses. Of these, 13% (406/3120) were fraudulent/suspicious based on research-informed criteria and were removed. Final analysis included 2,534 respondents, of which the majority (59.9%) reported hearing about the study from social media. Of the respondents, 49.5% were between 31-40 years, 39.5% were Black, Hispanic, or American Indian/Alaskan Native, and 45.9% had an annual household income below $50,000. Over half (55.3%) were assigned male, 40.4% were assigned female, and 4.3% were assigned intersex at birth. Transgender respondents made up 10.6% (n=267) of the respondents. In terms of sexual orientation, 54.1% (n=1371) reported being gay or lesbian, 30% (n=749) bisexual, and 15.8% (n=401) queer. A total of 756 (29.8%) respondents reported receiving a cancer diagnosis and among screen-eligible respondents, 66.2% reported ever having a Pap, 78.6% reported ever having a mammogram, and 84.1% reported ever having a colonoscopy. Over half of eligible respondents (58.7%) reported receiving Human Papillomavirus vaccinations.

**Conclusion:**

Study findings showcase effective strategies to reach communities, maximize data quality, and prevent the misrepresentation of data critical to improve health in SGD communities.

## Introduction

In the United States (U.S.), about 5% of the population identifies as Sexual and Gender Diverse (SGD) (1). SGD is an umbrella term used to describe individuals who are part of the LGBTQIA+ (lesbian, gay, bisexual, transgender, queer, intersex, asexual, and many other sexual orientations and gender identities) community. Despite SGD individuals representing a significant proportion of the population, there is limited research to identify and understand cancer prevention practices among SGD populations, both nationally and in NM.

NM is a minority-majority state, with 49.3% of the population being Hispanic or Latino, 11% being American Indian/Alaska Native, and 36.8% being non-Hispanic White (2). Additionally, about one third of New Mexicans report speaking a language other than English at home, with Spanish being the top language (3). NM is the third poorest state in the union with an average poverty rate of 16.2% compared to the U.S. average of 11.2% (4). Additionally, the Congressional Research Office considers 14 out of 33 NM counties to be Persistent Poverty Counties (poverty rates of 19.5% or greater), based on 1990 Census, Census 2000, and 2019 Small Area Income and Poverty Estimates (5). NM is also a very rural state, which can further contribute to disparities (6). Applying the most recent 2010 Rural-Urban Commuting Area (RUCA) codes, approximately 36% of New Mexicans live in rural areas, as defined by the Health Resources and Services Administration (HRSA) (7).

Recent data from the Williams Institute that reports results from the Gallup Survey (1) suggests that 4.5% of the New Mexico (NM) population identify themselves as Lesbian, Gay, Bisexual, or Transgender (LGBT) (8). Among NM SGD populations, 45% report male gender and 55% report female gender. The average age is 37.7 years and they are primarily Latino/a (49%) with 43% being white. Thirty nine percent report a high school education as their highest educational attainment and 14% report being uninsured. Finally, 26% of LGBTQ individuals in New Mexico report having an income less than $24,000 (8). Overall, the SGD communities in NM are relatively younger and primarily belong to groups that experience racial and socioeconomic inequities.

Collectively, the social determinants of health highlighted above can contribute to significant health disparities experienced by people living in NM, further highlighting the need to better understand population perspectives for designing and developing equity-oriented health interventions. For SGD populations, these determinants of health can intersect with their sexual and gender identities, and further exacerbate disparities (9, 10). For example, a transgender man who belongs to a group experiencing racial inequities and has a lower socioeconomic status would face greater barriers to access care as each one of these aspects of his identity is associated with lower access to care (11–13). The most recent (2014) epidemiological data from the NM Department of Health show differences in cervical and breast cancer screening uptake based on sexual orientation, but these data do not document disparities based on gender identity and for other types of cancer (2).

There is a dearth of data around NM SGD populations, especially for rural SGD populations. These populations can face different challenges than SGD populations in urban areas due to the complexity associated with socially conservative locals where they may not feel safe being “out” (14, 15). Juxtaposed with this notion, much of the extant SGD studies have been conducted in large urban cities, with these populations considered as “hard-to-reach” (16, 17). To address this gap, we sought to understand the determinants to cancer prevention practices (e.g. smoking, vaccination, cancer screening, among others) in order to develop future interventions. We proposed conducting a state-wide survey to better understand cancer prevention and control related practices among NM’s SGD populations. This paper presents the systematic and innovative strategies that were employed for reaching the NM SGD populations, with the goal of recruiting them to participate in a cross-sectional, purposive sampled, web-based survey.

The purpose of this paper is to present empirical data supporting the multi-pronged, low-cost, and systematic recruitment strategies to engage SGD communities in NM, a state that is largely rural, poor, and racially/ethnically classified as a “majority-minority” state. Additionally, we describe the characteristics of the study sample that was recruited using the aforementioned strategies. Data from the SGD communities were collected using online questionnaires which presented unique threats to sample and data validity. We also provide a detailed description on using research-informed strategies to detect fraudulent or suspicious responses and ensure data quality, and these methods can inform similar future efforts.

## Methods

### Recruitment Strategies

Recruitment for the survey took place from January to March 2021. To ensure state-wide reach, we focused on four methods (1): Every Door Direct Mail (EDDM) program, by the United States Postal Services (USPS) (2); advertisements on Google and Facebook (3); organizational outreach *via* emails to SGD-friendly businesses; and (4) personal outreach *via* flyers at clinical and community settings across NM. All flyers, ads, and emails contained QR codes (optical labels that contain links that can be accessed using a cellphone camera) and/or links to a survey in both English and Spanish.

**Figure 1:**
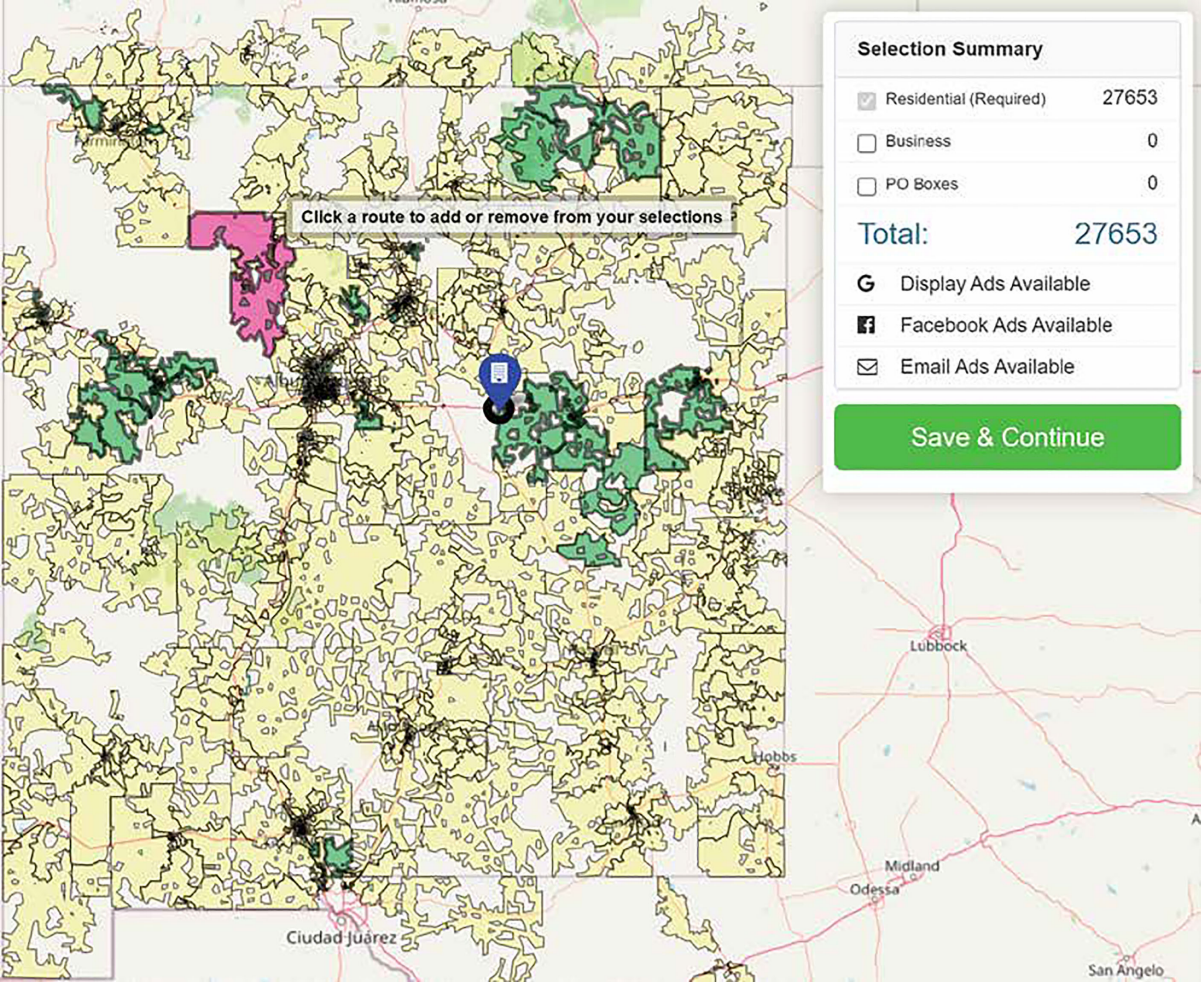
Online Mapping tool provided by Taradel to select specific mailing routes.

**Figure 2:**
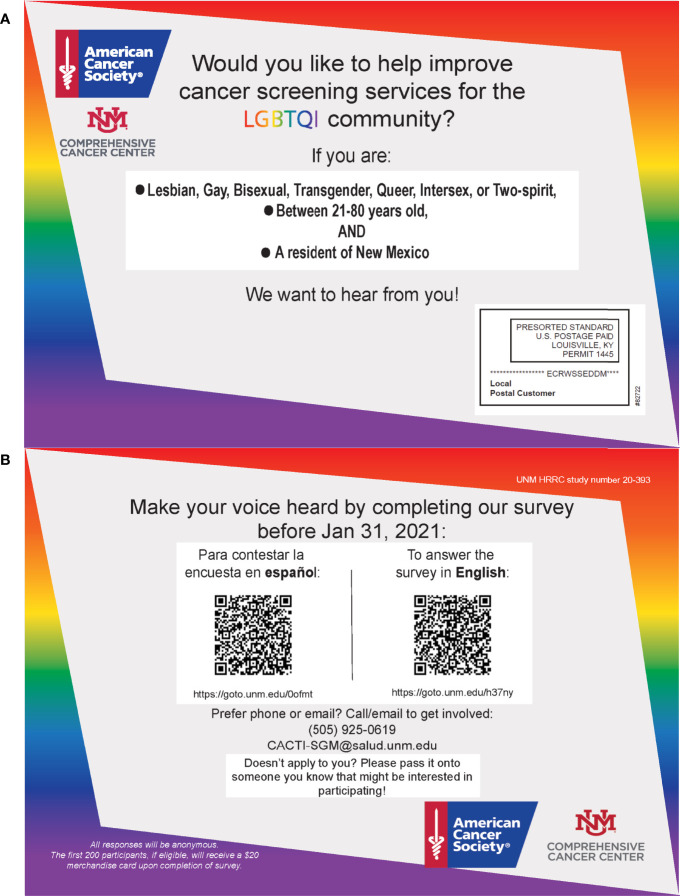
Flyers created for the study **(A)** Front of flyer, **(B)** Back of flyer.

The EDDM program by the USPS was the team’s primary method employed to reach SGD populations across the state. This program was originally designed to help businesses promote their products by mailing promotional materials to certain audiences in neighboring mailing routes (18). We worked with Taradel^®^ which is an affiliate of the USPS, that provides access and services in addition to EDDM (i.e. digital ads on Google, email outreach, and Facebook ads) (19). Taradel^®^ provides an online mapping tool (See **Figure 1**), which allowed the team to select mailing routes based on attributes such as residential or business addresses, household income, age, home ownership, gender, and presence of children, collated using data from the US Census Bureau (20). To inform the selection of the mailing routes, three criteria could be chosen from a drop-down menu, which then generated a heatmap overlay of mailing routes, indicating how each mailing route matched up with the specified criteria (**Figure 1**). We selected three criteria for this study: residential addresses, household income below 50,000, and age of residents 25 and older. These criteria allowed us to recruit individuals below the median income of NM of $50,000 per year (21) and around the age range at which cervical, breast, colorectal, and lung cancer screening tests are recommended (22–25). Based on the heatmaps generated with these criteria, we selected mailing routes across NM with at least a 50% match probability, oversampling where we had local knowledge of SGD friendly neighborhoods. This resulted in the selection of 61 mailing routes, with a total of 27,369 individual addresses. Flyers for the study were then sent to these addresses (See **Figure 2** for flyers).

**Figure 3:**
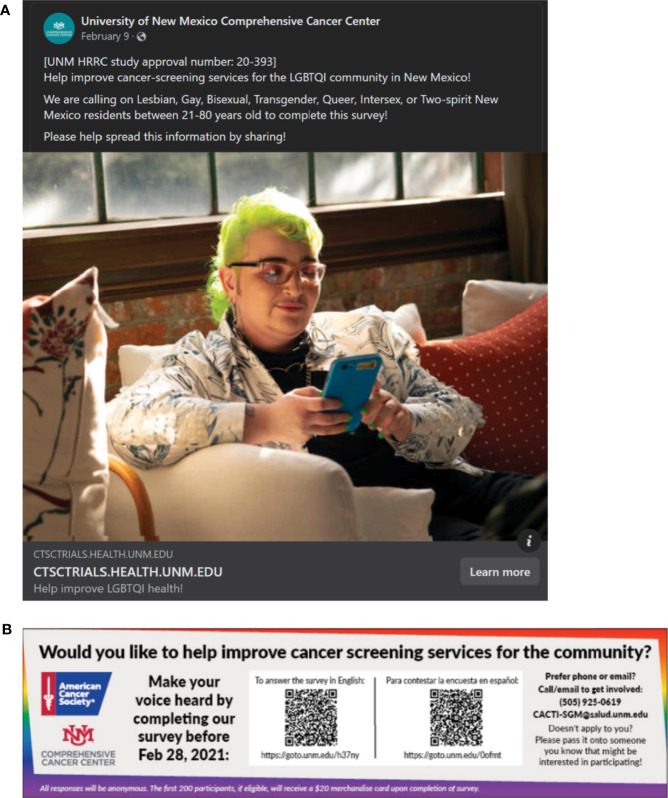
Facebook **(A)** and Google **(B)** ads created for the study.

The second method of using Google and Facebook ads was an added service provided by Taradel^®^, that overlapped with the 61 selected mailing routes for the flyers. With the help of their digital services department, we created digital ads that could be displayed through Google and Facebook Ads (See **Figure 3** for Google and Facebook Ads). To create these ads, we used publicly-available stock photos provided in The Gender Spectrum Collection (26) that represent transgender and nonbinary individuals. The Facebook Ads were created in collaboration with the UNM Comprehensive Cancer Center (UNMCCC), that allowed for the Ads to be hosted on UNMCCC’s Facebook page, lending credibility to the study recruitment. Both Google and Facebook Ads were disseminated using targeted e-mail and Facebook services provided by Taradel^®^.

The two other methods for reaching and recruiting individuals into the study relied on building organizational and interpersonal relationships with the NM community to help distribute the flyers and ads created in the previous strategies *via* email outreach. The NM OUT Business Alliance is an advocacy organization that seeks to “advance the common business interests, economic growth, and equality in the workplace and society for its LGBTQ members, businesses, and allies by providing educational, networking, and community building opportunities” (27). Toward their goal of cultivating certified suppliers, they offer a free certification for businesses and approximately 100 business in NM were advertised on their webpage in early 2021. We identified the publicly available email addresses for contacts listed on their online directory and sent them flyers.

We also partnered with the UNMCCC’s Office of Community Outreach and Engagement to promote the study outreach activities (e.g., regular newsletters sent out to members) and listservs. Similar efforts were undertaken through connections made with the NM Cancer Council (28), New Mexico State University’s Community Health Education Core, the University of New Mexico Health Sciences Center (UNM HSC) LGBTQ Collaborative (29), the UNM LGBTQ Resource Center (30), and Transgender Resource Center of New Mexico (31). Finally, laminated flyers (a requirement imposed due to the ongoing COVID-19 pandemic in order to make them easy to disinfect and clean) were placed in clinical and community settings (including the Southeast Heights Family Health Clinic (32) and the UNM Truman Clinic (33), (both providing care for the SGD populations in Albuquerque, NM) across the state, through study team relationships. Additionally, study team members searched for LGBTQIA+ Facebook groups throughout the state and sent them a message containing the flyer and Facebook ad, asking them to post to their pages.

### Survey Design and Distribution Methods

All study procedures received approval from University of New Mexico’s Human Research Protection Office (Study number 20-393). All surveys were managed using Research Electronic Data Capture (REDCap) tools hosted by the University of New Mexico (34, 35), that provides a secure, web-based application designed to support data collections for research studies. The surveys were designed in English and translated into Spanish by a certified translation specialist, and both English and Spanish versions were used for data collection. All surveys were pilot tested by the research team and a small group of volunteers prior to data collection. The surveys were designed with two specific objectives (1): to screen individuals that could participate in the survey, and (2) to elicit individual’s self-report of cancer screening and prevention practices.

The eligibility survey (**Supplementary Appendix 1A**, **1B**) queried about their age range, NM resident status, whether they were a member of SGD community, and how they heard about the study. If respondents answered that they were between 21-80 years old, were a resident of NM, and were members of the SGD community, they were considered eligible These criteria were used to specifically recruit individuals in the age range where cervical (21 – 65 years), breast (50 – 75 years), colorectal (45 – 36), and lung cancer (50 – 80 years) screenings are recommended (18, 19, 34, 35) and those who were members of the NM SGD community. All flyers, ads, and emails contained QR codes and/or links to a survey in both English and Spanish. All responses to the eligibility screener were reviewed by the study team weekly and duplicate emails, incomplete, and ineligible responses were removed.

All eligible respondents received the cancer prevention practices survey (**Supplementary Appendix 2A**, **2B**) which was adapted from the annual and lifetime surveys developed by the PRIDE study (37). We asked questions about demographics, body organs, physical health, Human Papillomavirus (HPV) vaccination, health care access, cancer screening practices (for cervical, breast, colorectal, and lung cancer) as well as what influenced these practices, and whether they would like to hear from us regarding study findings. Branching logic was applied depending on the age of the participant, their current body organs, whether they had been diagnosed with the type of cancer that was being asked about, and certain behaviors (i.e., if they had smoked at least 100 cigarettes in their lifetime for lung cancer screening). Two open-ended questions asked about anything else they would like to share about their health and if they had any additional comments about the survey. A total of 45-73 close-ended questions and two open-ended questions were included in the final survey. The survey was pre-tested and estimated to take 10-20 minutes, depending on branching logic.

To determine how many respondents were residents of rural counties, we asked the survey respondents to provide zip codes. We matched these with State-County-Tract FIPS codes, which were then matched with 2010 RUCA codes (7). The HRSA definition defines rural as all non-metro counties, tracts with RUCA codes between 4-10, and large metro tracts of at least 400 square miles in an area with a population density of 35 or less per square mile with RUCA codes 2-3. HRSA uses this definition to decide which areas are eligible for rural health funding (38). This definition of rural was then used to determine the percentage of respondents that provided a zip code in a rural area in NM.

The cancer prevention practices survey was sent to the eligible respondents either by email with a unique link to the survey or a paper copy of the survey mailed to their addresses, either in English or Spanish depending on their indicated preference in the eligibility survey. Emails that failed to deliver or bounced back were removed by default. We sent three reminders to those who requested the online version of the survey. The first 200 eligible participants who completed the survey received $20 provided in acknowledgement of the respondent’s time and expertise *via* Amazon gift card codes.

### Ensuring Data Quality for Survey Research

Having respondents complete the eligibility survey before the cancer prevention practices survey allowed researchers to exclude social bots [software that is programmed to enter many responses in order to receive incentives (39)], duplicates, and ineligible respondents. Despite these phased approaches, there were instances of duplicate qualitative responses in the survey data, which triggered an additional search for strategies to ensuring data quality. We followed the detailed and systematic guidance provided by Pozzar and colleagues (40), in ensuring quality of data collected through social media research. Based on guidance from this research, we defined and operationalized four fraudulent and 17 suspicious criteria (details on the list of fraudulent and suspicious criteria are provided in **Table 1**). We removed responses with one fraudulent criterion or three or more suspicious criteria.

**Table 1:** Fraudulent and suspicious criteria applied to ensure data quality.

Fraudulent criterionN = 489 removed		Justification
Duplicate emails	1	These emails were duplicated in the responses for how individuals would like to hear about the study or how they would like to receive their merchandise card, indicating they were from the same respondent.
Exact matches in qualitative responses greater than 3 words	2	These responses were under the two qualitative questions we asked on the survey and there were several duplicate responses in these fields across respondents.
Non-US zip code	3	Non-US zip codes indicated that these respondents were not part of our desired study population and therefore likely bots.
Respondent that they were heterosexual and cisgender	4	These individuals were not part of the study population we were wanting to query and were therefore removed from the analysis.
Suspicious criterion N = 97 removed		
Non-NM zip code	1	One criterion for inclusion in this study was that the respondent was a resident of New Mexico. However, we felt that individuals that were residents of New Mexico could be receiving their mail in a location outside of the state, so we made the decision to make this criterion suspicious rather than fraudulent.
Height greater than or equal to 7 feet or less than 4 feet	2	Using the Body Mass Index table, we identified the range of heights that were likely, and considered heights outside of that range suspicious (41).
Weight less than 90 pounds	3	Using the Body Mass Index table, we identified the low end of weight considered and considered weights under than suspicious (41).
Nonsensical qualitative responses	4	Responses were considered suspicious if they didn’t make sense in response to the question asked. Examples of these responses include “Establish a federal bullying task force” in response to the question “Is there anything else you would like to share with us about your health or well-being?” and “Provide emergency shelters and support services” to the same question.
Respondent indicated that they were a transgender woman and AFAB	5	These responses were considered suspicious because the term “transgender” refers to someone whose gender does not align with the sex assigned at birth. Therefore, if a respondent were AFAB and identified as transgender, they would be a transgender man or one of the other nonbinary options, not a transgender woman.
Respondent indicated that they were transgender man and AMAB	6	These responses were considered suspicious because the term “transgender” refers to someone whose gender does not align with the sex assigned at birth. Therefore, if a respondent were AMAB and identified as transgender, they would be a transgender woman or one of the other nonbinary options, not a transgender man.
Respondent indicated that they were a cisgender woman and AMAB	7	These responses were considered suspicious because the term “cisgender” refers to someone whose gender aligns with the sex they were assigned at birth. Therefore, if a respondent were AMAB and identified as cisgender, they would be a cisgender man and not a cisgender woman.
Respondent indicated that they were a cisgender man and AFAB	8	These responses were considered suspicious because the term “cisgender” refers to someone whose gender aligns with the sex they were assigned at birth. Therefore, if a respondent were AFAB and identified as cisgender, they would be a cisgender woman and not a cisgender man.
Respondent indicated that they were a masculine gender (cisgender man, transgender man, or man) and a lesbian	9	These responses were considered suspicious because the term “lesbian” refers to a woman who is attracted to other women. Therefore, if someone’s gender is masculine, it is unlikely that they also identify as a lesbian.
Respondent indicated that they were heterosexual and another sexual orientation other than asexual	10	The term “straight” indicates that one is attracted primarily to members of the opposite gender, and is generally exclusive of other orientations. This is not to say that straight individuals are not sometimes attracted to people of the same sex or even have sex with them, but that they do not generally also identify as a member of the SGD community (42). However, the sexual orientation of asexual can exist on a spectrum and refers to individuals who have little or no attraction to others, and it does not indicate what gender(s) they may be either sexually or romantically attracted to.
Respondent indicated that they were AMAB and had a vagina and a penis	11	These responses were considered suspicious because genital gender affirmation surgeries for individuals who are AMAB usually consist of a vaginoplasty (creation of a vagina) that is created through some form of penile inversion procedure, where the lining of the vaginal canal is created from the skin of the penis (43). Therefore, if an individual AMAB had genital gender affirmation surgery that consisted of the creation of a vagina, they would no longer have a penis as part of the vaginoplasty is inversion of the penis.
Respondent indicated that they were AMAB and had a uterus	12	These responses were considered suspicious because, while the technology of uterine transplantation is being developed and looks promising, this is currently not approved as part of genital gender affirmation surgeries and therefore someone who was AMAB would likely not have a uterus.
Respondent indicated that they were AMAB and had a cervix	13	These responses were considered suspicious because, while the technology of uterine transplantation is being developed and looks promising, this is currently not approved as part of genital gender affirmation surgeries and therefore someone who was AMAB would likely not have a cervix (44).
Respondent indicated that they were AMAB and had ovaries	14	These responses were considered suspicious because, much like uterine transplantation, ovarian transplantation is currently not approved as part of genital gender affirmation surgeries and therefore someone who was AMAB would likely not have ovaries (45).
Respondent indicated that they were AFAB and had a uterus and no vagina.	15	These responses were considered suspicious because, in genital gender affirmation surgery for individuals AFAB, a vaginectomy (removal of the vaginal canal) is never performed if a total hysterectomy (removal of the uterus along with the cervix) has not already been performed. This is because the vaginectomy would make it impossible to screen for cancer (46).
Respondent indicated that they were AFAB and had a cervix and no vagina.	16	These responses were considered suspicious because, in genital gender affirmation surgery for individuals AFAB, a vaginectomy (removal of the vaginal canal) is never performed if a hysterectomy (removal of the uterus along with the cervix) has not already been performed. This is because the vaginectomy would make it impossible to screen for cancer (46).
Respondent indicated they were AFAB and had a prostate	17	These responses were considered suspicious because a prostate is not present in individuals who were AFAB and prostate transplants are not currently an option for masculinizing genital gender affirmation surgery (26).

AMAB = Assigned Male at Birth; AFAB = Assigned Female at Birth.

For fraudulent criteria, we considered responses that had (1) mismatched email addresses noted in answers from the same respondent (2); exact matches in qualitative responses greater than three words (3); reported non-US zip codes; and (4) reported to be heterosexual and cisgender. The 17 suspicious criteria broadly fell under four categories of responses with (1): non-NM zip codes (2); Height greater than or equal to 7feet or less than 4 feet and weight less than 90 pounds (3); nonsensical qualitative responses; and (4) incongruence between SAAB (sex assigned at birth), gender identity, sexual orientation, and current body organs. This last category considered suspicious were based on definitions of terms (i.e., “transgender”, “cisgender”, “straight” and “lesbian”) (42, 47) as well as current practices in genital gender affirmation surgeries (43–46).

## Results

### Reaching the NM SGD Populations

**Figure 4:**
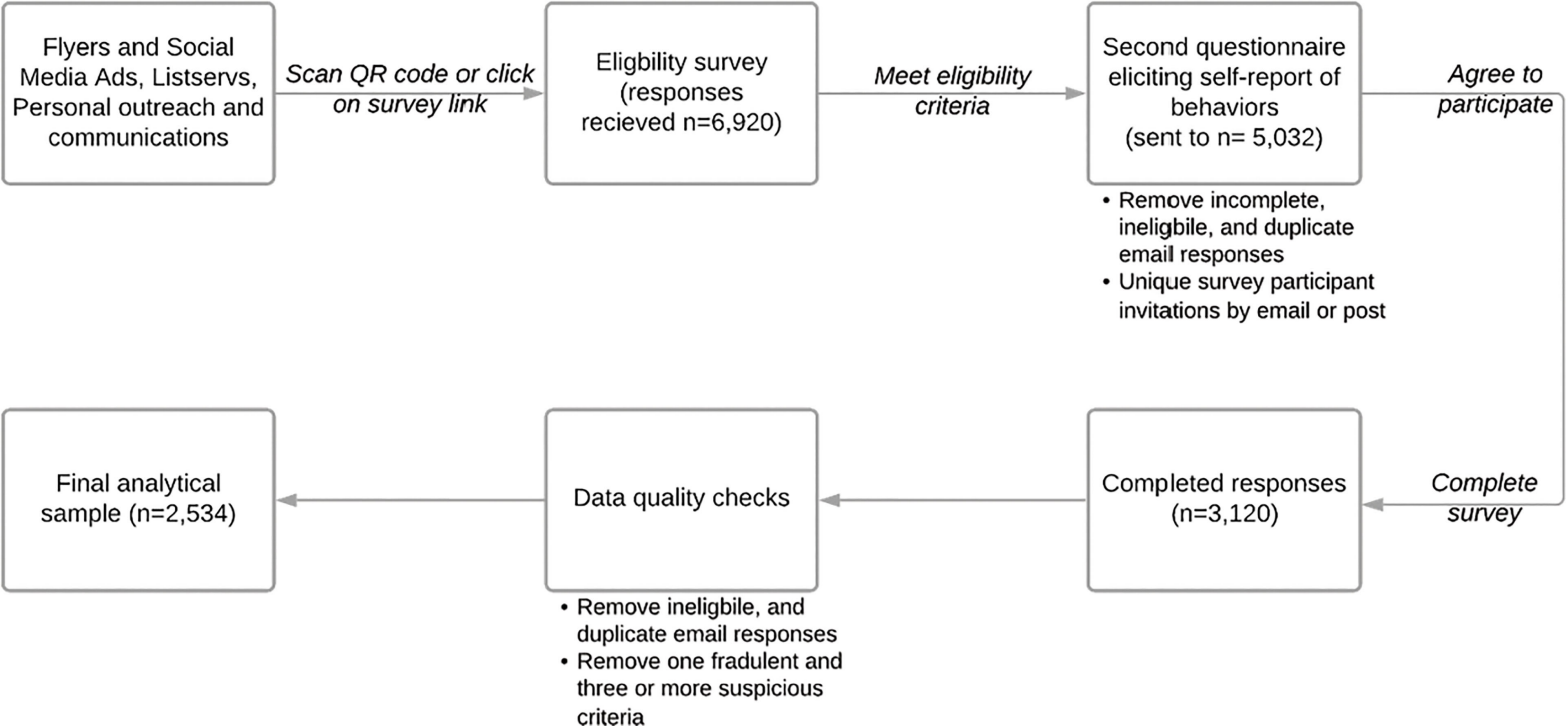
Study Flow.

**Figure 5:**
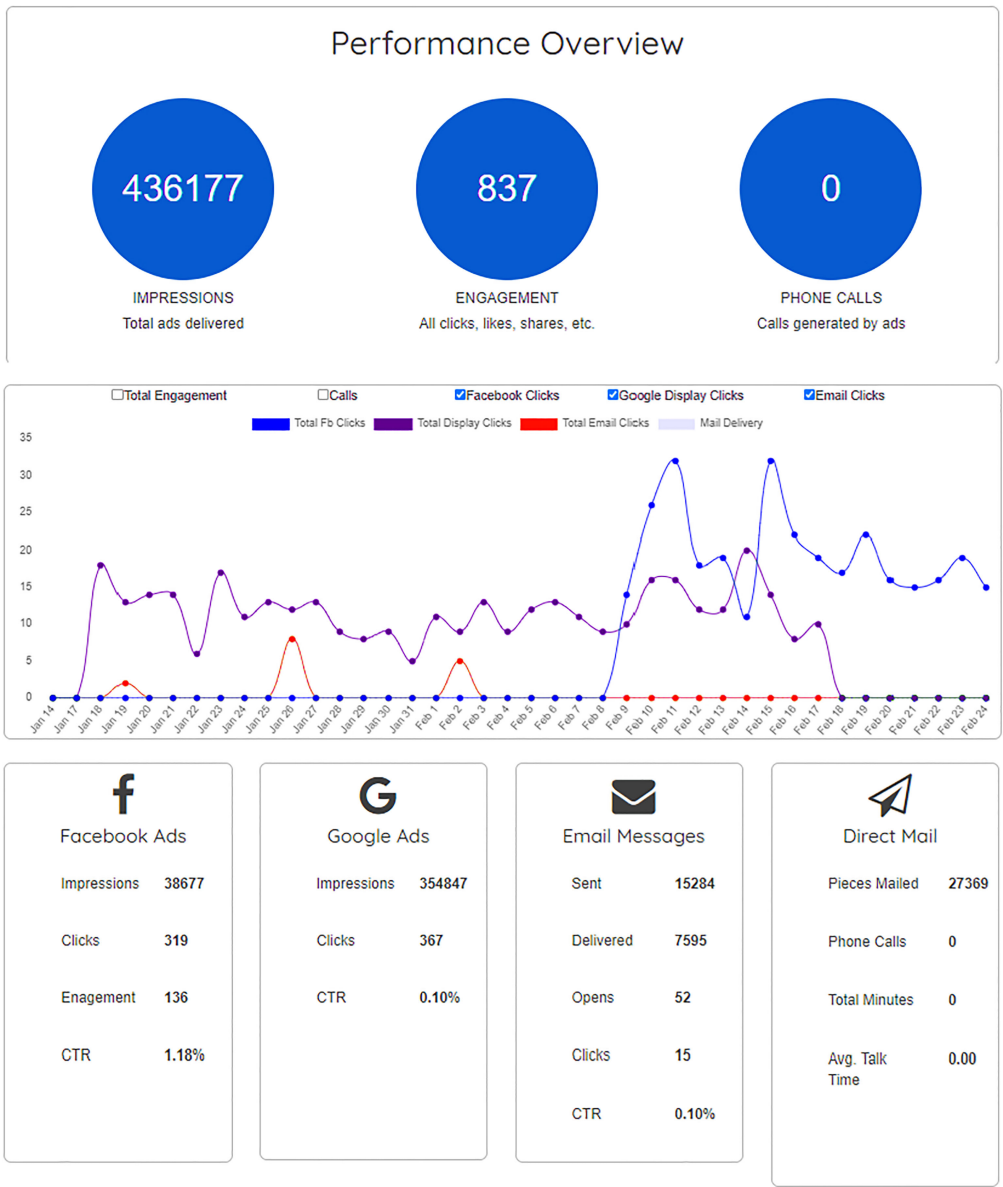
Detailed data on the Facebook Ads, Google Ads, Emails, and Direct Mail from Taradel.

The strategies of recruitment and data quality assurance detailed above collectively contributed to the recruitment of the overall sample of 2,534 respondents included in the analysis for this study. The overall study flow is shown in **Figure 4**. With the EDDM program, we selected over 61 mailing routes across the state where 27,369 flyers (highlighted as Direct Mail in **Figure 5**; this figure was altered in the form of color change to increase visibility and accessibility) were distributed to the residential addresses. The EDDM vendor provided detailed data (see **Figure 5**) on the overlapping strategy for the targeted Google Ads and Facebook Ads to residents on these mailing routes that collectively led to 393,523 impressions [i.e. how many times an ad was displayed on a person’s screen for Facebook (48) or on a search result page for Google (49)], resulting in 686 clicks (i.e. how many times a person clicked on the ad). The same vendor also sent 15,284 emails, of which 7,595 were delivered, 52 were opened, and 15 people clicked on the content inside the email. Based on these numbers, we believed we reached a total of 436,177 individuals, with the total cost of 3¢ per person reached (i.e. 436,177 impressions) or approximately $6 per complete response (i.e. 2,534 responses included in the final analyses) including incentives for the first 200 respondents (50).

Although it was difficult to estimate the reach of the other strategies that utilized the team members and organizational connections in the community, we asked survey respondents how they heard about the study in both the eligibility survey and the cancer screening practices survey. **Tables 2.1**, **2.2** presents detailed data on these responses; overall, however, respondents noted social media as the most common way they heard about the study. In seven weeks of data collection, we received a total of 6,920 responses on the eligibility survey (English:6,139; Spanish:781). Of these, 27% (n=1,888) were duplicate, incomplete, or ineligible responses with the majority (98%) being duplicate responses that were likely sent from bots. We sent 5,032 unique survey links and mailed five paper surveys. No paper surveys were returned, and we received 3,120 complete online responses (English:2,811; Spanish:309), indicating a response rate of 62%. Survey responses were received from 163 unique NM zip codes and 18% reported living in rural areas in NM as defined by the HRSA (38).

**Table 2.1:** Survey respondents from eligibility screener and pre-cleaned main survey responses.

Participant’s response to the question“How did you hear about this survey?”	Number of Responses (n)	Percentage of Responses* (%)
**Eligibility survey (n = 6,920)**		
Social media (Facebook/Twitter)	5,249	75.9
Email	2,582	37.3
Mailed flyer	2,305	33.3
Family/friend/colleague	2,526	36.5
Other**	13	0.2
Missing	24	0.3
***Main survey (n = 3,120)***		
Social media (Facebook/Twitter)	1866	59.9
Email	1299	41.7
Mailed flyer	470	15.1
Family/friend/colleague	232	7.5
Other**	6	0.2
Missing	8	0.3

*Respondents were given the option to select all that apply

**“Other” responses included: LGBTQ collaborative, PFLAG Silver city, NM, Pop-up

[Ads], listservs, flyers, newsletters, and don’t remember

**Table 2.2:** Survey respondents from final data.

Participant’s response to the question “How did you hear about this survey?” (n=2,534)	Sex Assigned at Birth n (%) *			Gender n (%) *		Sexual orientation n (%) *			
	Female (n=1025)	Intersex (n=108)	Male (n=1401)	Transgender/non-binary (n=508)	Cisgender (n=2026)	Lesbian/Gay (n=1371)	Bisexual (n=749)	Queer (n=401)	Straight (n=13)
Social media (Facebook/Twitter)	716 (69.9)	44 (15.1)	863 (61.6)	253 (49.9)	1370 (67.6)	878 (64.0)	505 (67.4)	238 (59.4)	2 (15.4)
Email	367 (35.8)	44 (15.1)	567 (40.5)	238 (46.9)	740 (36.5)	548 (40.0)	467 (62.3)	155 (38.7)	8 (61.5)
Mailed flyer	93 (9.1)	18 (16.7)	133 (9.5)	47 (9.3)	197 (9.7)	126 (9.2)	86 (11.5)	31 (7.7)	1 (7.7)
Family/friend/colleague	72 (7.0)	10 (9.3)	88 (6.3)	58 (11.4)	112 (5.5)	80 (5.8)	46 (6.1)	37 (9.2)	7 (53.8)
Other**	4 (0.4)	0 (0)	2 (0.1)	2 (0.4)	4 (0.2)	4 (0.3)	1 (0.1)	1 (0.2)	0 (0)

* Respondents were given the option to select all that apply.

** “Other” responses included: LGBTQ collaborative, PFLAG Silver city, NM, Pop-up [Ads], listservs, flyers, newsletters, and don’t remember.

### Data Quality Check

As shown in **Table 1**, of the 3,120 responses, 16% (n=489) were fraudulent and were removed. Of the remaining 2,631 respondents, 4% (n=97) met three or more criteria for being deemed suspicious and were excluded from analysis, leaving a total of 2,534 responses included in analysis. Thirteen percent (n=330) met two or more of the suspicious criteria and 30% (n=788) met at least one suspicious criterion, neither of which were grounds for removal from analysis.

### Overall Characteristics of the Population Sample

**Table 3** provides detailed data about the characteristics of the survey respondents included in the final analysis (n=2534). Over half of the respondents (55.3%) were assigned male at birth, 40.4% were assigned female at birth, and 4.3% were assigned intersex at birth. Transgender respondents made up 10.6% (n=267) of the study sample, while 46.1% of respondents reported being cisgender men and 33.8% reported being cisgender women. In terms of sexual orientation, 54.1% (n=1371) reported being gay or lesbian, 30% (n=749) bisexual, and 15.8% (n=401) queer. Around 85% of the respondents were between the age of 21-40 years. Black, Hispanic, and American Indian/Alaska Native respondents comprised 39.6% of the respondents. Regarding education, income, and employment, 43.7% reported high school or some vocational training as their highest level of education, 45.9% had an annual household income below $50,000, and 19% reported being unemployed.

**Table 3:** Demographic characteristics of the population sample.

Characteristic	n (%)
**Sex assigned at birth**	
Female	1025 (40.4)
Intersex	108 (4.3)
Male	1401 (55.3)
**Gender**	
Cisgender man	1169 (46.1)
Cisgender woman	857 (33.8)
Non-binary	241 (9.5)
Transgender man	126 (5.0)
Transgender woman	141 (5.6)
**Sexual orientation**	
Bisexual	749 (30.0)
Lesbian/gay	1371 (54.1)
Queer	401 (15.8)
Straight	13 (0.5)
**Age**	
21-30y	901 (35.5)
31-40y	1255 (49.5)
41-50y	308 (12.2)
51-60y	52 (2.1)
61-70y	14 (0.6)
70-80y	4 (0.2)
**Race**	
American Indian or Alaska Native	75 (3.0)
Black, African American, or African	299 (11.8)
Hispanic	628 (24.8)
White	1324 (52.2)
Other	135 (5.3)
Missing	73 (2.9)
**Education**	
High school or less	239 (9.4)
Some college or vocational training	869 (34.3)
College and/or advanced degree	1411 (55.7)
Missing	15 (0.6)
**Income**	
$0-$30,000	314 (12.4)
$30,000-$50,000	849 (33.5)
$50,000-$70,000	872 (34.4)
$70,000+	495 (19.5)
Missing	4 (0.2)
**Employment**	
Employed	1990 (78.5)
Unemployed	482 (19.0)
Missing	62 (2.4)
**Rural/Non-Metropolitan *vs* Metropolitan**	
Rural/Non-metropolitan	449 (17.7)
Metropolitan	1675 (66.1)
Missing	410 (16.2)

In relation to their access to health care (shown in **Table 4**), 22.3% (n=564) reported being uninsured, 27.3% (n=691) reported not having a place to go to for routine check-ups, and 42.2% (n=1069) reported unnecessary delays in getting medical care in their lifetime. Overall, 66.3% reported having a primary care provider, however, in the past 12 months, 30% had not seen their provider. In the past 12 months, 47% reported having no insurance, 34% had trouble finding a provider, and 55.5% reported being unable to obtain care. Overall, 31.1% reported being denied or given lower quality medical care in the past 12 months. Among the 797 respondents that were asked about cervical cancer screening, 66.2% (n=528) of pap smear-eligible respondents reported ever being screened with Pap smears. Similarly, 78.6% (n=55) of the 70 mammogram-eligible respondents reported ever receiving a mammogram, 84.1% (n=53) of the 63 colorectal cancer screening-eligible respondents had ever been screened for colorectal cancer, and 33.3% (n=5) of the 15 lung cancer screening-eligible respondents reported ever being screened for lung cancer. A total of 756 (29.8%) people reported receiving a cancer diagnosis. Of respondents who were eligible (i.e. below 46 years old) for the HPV vaccine (n=2379), 58.7% reported receiving a vaccine for HPV.

**Table 4:** Characteristics of the population in terms of access to care and cancer prevention practices.

Characteristic	n (%)
**Insurance**	
Yes	1911 (75.4)
No	564 (22.3)
Don’t know	47 (1.9)
Missing	12 (0.5)
**Place to go for routine check up**	
Yes	1702 (67.2)
No	691 (27.3)
Don’t know	115 (4.5)
Missing	26 (1.0)
**Unnecessary delay in getting medical care**	
Yes	1069 (42.2)
No	1346 (53.1)
Not applicable	96 (3.8)
Missing	23 (0.91)
**Primary care physician**	
Yes	1681 (66.3)
No	767 (30.3)
Don’t know	63 (2.5)
Missing	23 (0.9)
Seen primary care in past 12 months (n=1681)*	
Yes	1147 (68.2)
No	504 (30.0)
Don’t know	15 (0.9)
Missing	15 (0.9)
**Uninsured in past 12 months**	
Yes	1277 (50.4)
No	1191 (47.0)
Don’t know	53 (2.1)
Missing	14 (0.6)
**Trouble finding provider past 12 months**	
Yes	861 (34.0)
No	1355 (53.5)
I haven’t tried	267 (10.5)
Don’t know	34 (1.3)
Missing	17 (0.7)
**Unable to obtain care in the past 12 months**	
Yes	1010 (39.9)
No	1407 (55.5)
Not applicable	95 (3.7)
Missing	22 (0.9)
**Denied or given lower quality medical care in the past 12 months**	
Yes	787 (31.1)
No	1621 (64.0)
Not Applicable	115 (4.5)
Missing	11 (0.4)
**Previous cancer diagnosis**	
Yes	756 (29.8)
No	1760 (69.5
Don’t know	0 (0)
Missing	18 (0.7)
**Ever received cancer screening**	
Cervical (n=797)	528 (66.2)
Breast (n=70)	55 (78.6)
Colorectal (n=63)	53 (84.1)
Lung (n=15)	5 (33.3)
HPV Vaccination (n = 2379) **	
** Yes**	1396 (58.7)
** No**	862 (36.2)
** Don’t know**	112 (4.7)
** Missing**	9 (0.4)

*Only asked for the respondents that said they had a primary care provider.

**Calculated using eligible respondents (under 46 years old).

A total of 1752 (69%) respondents confirmed that they would like to hear from us regarding study findings and 371 (47%) respondents with a cervix answered that they would be interested in participating in a focus group about cervical cancer screening. On the open-ended questions, many respondents expressed their appreciation of the inclusive nature of the survey in queries around sexual orientation and gender identity (SOGI) status. For example, a respondent stated,

“Appreciated the survey trying really hard to be gender and orientation inclusive! I think fat shaming comes up for a lot of queer and trans folks as well so I appreciated that note…”

Other participants noted the importance of such a study and the resources for their community, stating:

“Thank you for doing this study! It’s very important and I appreciate that you are doing this work … Thank you for conducting a survey on such an important topic and for providing excellent resources at the end. I didn’t know about some of these resources and hope to reach out for myself and share them with others”.

## Discussion

Reaching SGD populations has been a challenge for the field of health research and it is crucial to find ways to mitigate this issue, especially in regards to research on healthcare and health behaviors (51, 52). This paper highlights the variety of methods (i.e. flyers, social media, organizational, and personal outreach) available to the cancer research community to reach diverse populations. We also present additional considerations to ensure that survey questions are asked in a respectful, culturally appropriate manner, which is especially important when surveying populations that have been marginalized. Finally, as opposed to conceptual guidance, we present detailed guidance on how we applied strategies, informed by prior social media research (40, 51, 53), to ensure data quality for SGD research.

To our knowledge, this is one of the few studies to employ EDDM to distribute flyers for an online, population-based health survey (54, 55). This survey’s ability to reach individuals from rural areas of the state indicates that utilizing social media and USPS is likely to be an effective method for states with significant rural populations when seeking diverse engagement. Among younger SGD populations, social media has been used successfully to engage and recruit participants (56). However, its use remains limited in engaging rural and adult populations (15), both of whom remain significantly underrepresented in population-based SGD research. Innovative and systematic efforts are needed in order to develop meaningful interventions that address the healthcare needs of marginalized rural SGD individuals (57). In this study, more than 85% of respondents were between 21-40 years old, so additional methods may be needed to support recruitment for the over-40 SGD community. Some studies have had success with crowdsourcing platforms (58) and other methods, such as referrals from current participants and review of electronic medical records to identify eligible participants (59). However, social media provides an important opportunity to engage the SGD community with the potential for future public health interventions and behavioral research. In previous public health studies, impressions have been considered to be equivalent to reaching populations, while clicks indicated the actual behavior of the person (53, 60).

Advocates for promoting SGD research express that these populations are eager to participate in research, especially if their participation can benefit their community (57). This might partly explain the overwhelming response to our recruitment strategies (i.e. 62% response rate). The survey took anywhere from 10-20 minutes to complete and had a monetary incentive, which may have further influenced the response rate. We surmise that another reason for the high number of responses was because people spent most of their time at home during a major wave of the COVID-19 pandemic that occurred in early 2021, which was when we started recruiting participants. However, other research done during the COVID-19 pandemic provides contrasting data. While survey-based research increased significantly during the pandemic, people’s responses to surveys tended to decline. The Census found that responses to surveys initially increased at the start of the pandemic, but declined and remained lower than non-pandemic years around the summer of 2020 (61). It has been suggested that the three-fold increase in social media-based recruitment and online survey administration during the pandemic likely led to “survey fatigue.” (62)

Another potential justification for the large number of respondents may be that we ensured an appropriate inquiry into SOGI status for the respondents. As mentioned above, the comments left at the end of the survey indicated appreciation for the respectful ways in which the questions were asked, and gratitude that the research was being conducted in the first place. These responses underscore the importance of gender expansive, non-heteronormative language in both research and healthcare. Using gendered and heteronormative language can alienate SGD populations and lead to reduced care-seeking practices (63). This highlights the need for using gender expansive language in educational materials as well as in intake forms and when speaking to patients and other healthcare providers.

Social media and other internet-based methods are considered to be cost-effective and often provide greater reach to the populations of interest (64, 65). However, they do present issues with people enrolling more than once, social bots, and respondents who do not fit the eligibility criteria (40, 65). Additional strategies to manage this type of data collection and prevent/detect fraudulent responses are necessary (40). We created consistent criteria with which to evaluate responses in order to determine authenticity and mitigate fraud. We also filtered out-of-country zip codes, along with removing any users with identical answers to open-ended questions. These methods proved to be efficient for removing many of the most obviously fraudulent responses. Less apparent instances of fraud were difficult to detect, but employing parameters such as height and weight, as well as incongruent answers to SAAB and gender, to deem a response fraudulent or suspicious worked well in this study’s data cleaning process. These criteria, however, were challenging to apply for the intersex population in this study and may partially explain their higher proportion (4.3%) in the study sample compared to rest of the population (1.7%) (66).

Compared to the Williams Institute, there were slightly more respondents in our study who reported male gender, with 51% of study respondents reporting male gender versus 45% from the Williams Institute. There were also more white respondents in our study (52.2% *vs* 43%). Respondents in our study had higher educational attainment with 55.7% of them reporting post-graduate schooling versus 16% reported from the Williams Institute (2). Access to care for this study population reflects previous studies (67), in that it shows lower insurance rates among SGD populations as well as a lower number of SGD individuals reporting having a primary care provider. The uninsured rate in this study population (22.0%) higher than the state average for adults 19 – 64 years old (18.8%) (68, 69), and the percentage of individuals with a primary care provider (66.3%) is also lower than the state average of 71.5% (70). These data represent a troubling trend around access to care for SGD populations which can lead to decreased rates of cancer screening services as well as treatment for other serious conditions (71). This disparity in access to care is likely multifactorial with stigmatization and discrimination in healthcare (72, 73) as well as in the workplace being a large contributor (74). Stigmatization and discrimination in healthcare can lead to SGD individuals’ decreased interaction with the healthcare system (71, 72), while discrimination in the workplace can lead to lower insurance rates among this population (74). Additionally, results gathered by this study illustrate the impact that COVID-19 has had on the healthcare field and the accessibility of services for all populations (75)., showing decreased access to care over the previous 12 months. This impact was more pronounced among populations that experience inequities due to their rural residence, since a common solution for access to care during the pandemic was telehealth, which, further exacerbated the digital divide in these communities and a considerable drop in cancer screening (36, 76).

About 69% of the survey respondents expressed interest in staying engaged and finding out more about the study. Respectful of this enthusiasm and in line with the guidance from the cancer research community working with SGD populations, we are committed to centering the study findings and future research within community perspectives (40, 63, 64). We utilized the Community Engagement Studio (CES) as a strategy to build relationships within the community. The CES is a consultative model that allows for research specific consultations from community members, often led by the Clinical Translational Center (60, 61). We conducted a Community Engagement Studio (CES) with 11 members from the NM SGD community. Conversations with the community members helped gather insights on communication strategies, future research endeavors, and opportunities to collaborate with the SGD community in NM. When asked about engagement, studio participants described their preferences in not using the term “sexual gender diverse communities” because they did not identify with the term, and instead preferred being referred to as the “LGBTQ+” community. Several mentioned the need for quick, relevant communication materials to engage the community (e.g., a 60-90 second video *via* Tik-Tok, YouTube, or Instagram/Facebook stories, or an Instagram/Twitter infographic with a “link in bio” or QR code). The group mentioned engaging young adults early in prevention, educating clinicians on the importance of inclusive language, collaborating with clinics that have direct trans and queer healthcare provision experience, and addressing the bias against the community from the medical providers. The majority of the community experts expressed interest in continued future discussions and we hope to engage them as a study-specific advisory council for gathering guidance on future research endeavors.

As with most research, this study is subject to certain limitations. These limitations primarily result from the use of a purposive sampling technique, volunteer bias, and the use of the internet to both recruit participants and to administer the survey. Purposive sampling, a process in which researchers pick particular segments of the population to recruit for a study, is prone to researcher bias and can result in a lack of generalizability (77). Such a strategy was suitable, because this was a pilot study aimed at gaining a preliminary understanding of the cancer screening practices of the SGD community in NM. Using purposive sampling to determine whether or not a perceived issue needs more research and resources devoted to it is widely regarded as a reasonable and effective use of this sampling method despite its inherent limitations (77). Even though two of our four methods relied on personal and organizational outreach, these relationships (i.e., community outreach and engagement offices in cancer centers, and LGBTQ-friendly businesses, among others) exist in several academic-community environments and could be leveraged for similar research studies. We also did not send survey links to people who requested paper surveys because they only provided a postal mailing address in the eligibility. Similarly, we did not attempt to send a paper survey to respondents who did not complete the online survey because they did not provide a postal mailing address. Older people are less likely to respond to web-based surveys (78) and it is likely that we could have had a higher proportion of older LGBTQIA+ adults with the paper survey.

Volunteer bias may have also played a role in our research, as it does in most survey-based studies. The pool of respondents was undoubtedly limited by the fact that participants were asked to answer questions regarding their sexual orientation and gender identity. Many people are not willing or not able to be open with such intimate information. This also applies to other portions of the survey, such as questions that asked about anatomy, cervical cancer screening, or colorectal cancer screening. Many people are not comfortable discussing such topics, and thus those who chose to participate in this survey were likely only a subset of the SGD population in NM. Furthermore, the recruitment flyers advertised a monetary incentive, which may have biased participation towards those experiencing financial need.

Finally, using the internet for reaching these populations can be limiting, as internet access in NM is poor, ranking 45th in the country (18). Internet-based research also typically reaches individuals of higher socioeconomic status and a younger population sample, which may not be representative of the target population (79). Younger people tend to use technology more than older people, which aligns with our findings that over 80% of survey respondents were under 40 years old (80). We believe that some of these barriers were overcome by mailing out flyers. Flyers however, were only delivered to individuals with residential addresses. This is especially pertinent information to take into account when doing research with the SGD community whose members, on average, experience homelessness more than twice as much as their cisgender, heterosexual counterparts (81).

## Conclusion

To reach state-wide SGD communities and engage them in population-based research, innovative and systematic efforts are needed. Social media and postal flyers may provide successful recruitment opportunities with potential to use these methods for future public health interventions in these populations. When using the online surveys to maximize reach, additional strategies to manage these data and prevent/detect fraudulent responses are needed. While time-intensive, the methods in this study were an effective way to ensure accurate data and to narrow down the responses to include only genuine answers that each represented one individual. Findings from this paper have the potential to maximize data integrity and prevent misrepresentation of health data for these communities.

## Data Availability Statement

The raw data supporting the conclusions of this article will be made available by the authors, without undue reservation.

## Ethics Statement

The studies involving human participants were reviewed and approved by University of New Mexico’s Human Research Protection Office (Study number 20-393). The participants provided their written informed consent to participate in this study.

## Author Contributions 

PA, conceptualized the article and PA, KM, and TJ drafted the manuscript. DK, VP, BT, EW, MM, SM, MK, and PM participated in the workgroup discussions that informed the manuscript making substantial contributions to the design and analysis presented in the paper and revised it critically for important intellectual content. All authors contributed to the article and approved the submitted version.

## Funding

The research reported in this paper was funded by an Internal Research Grant (PI: PA) from the American Cancer Society 131567-IRG-17-178-22-IRG (PI: Ozbun, M.) and was partially supported by UNM Comprehensive Cancer Center Support Grant NCI P30CA118100 and the Behavioral Measurement and Population Sciences shared resource. The Community Engagement Studio received support from the University of New Mexico’s Clinical Translational Science Center.

## Conflict of Interest

The authors declare that the research was conducted in the absence of any commercial or financial relationships that could be construed as a potential conflict of interest.

## Publisher’s Note

All claims expressed in this article are solely those of the authors and do not necessarily represent those of their affiliated organizations, or those of the publisher, the editors and the reviewers. Any product that may be evaluated in this article, or claim that may be made by its manufacturer, is not guaranteed or endorsed by the publisher.

## Supplementary Material

Supplementary Appendix 1A Eligibility survey in English.

Supplementary Appendix 1B Eligibility survey in Spanish.

Supplementary Appendix 2A Cancer prevention practices survey in English.

Supplementary Appendix 2B Cancer prevention practices survey in Spanish.

## Abbreviations

EDDM, Every Door Direct Mail; HPV, Human Papillomavirus; HRSA, Health Resources and Services Administration; LGBTQIA, Lesbian, Gay, Bisexual, Transgender, Queer, Intersex, Asexual + many other sexual orientations and genders; REDCap, Research Electronic Data Capture; RUCA, Rural-Urban Commuting Area; SAAB, Sex Assigned at Birth; SGD, Sexual and Gender Diverse; SOGI, Sexual Orientation and Gender Identity; UNMCCC, University of New Mexico Comprehensive Cancer Center; USPS, United States Postal Service.
