# Supplementary material for: Reaching the “Hard-to-Reach” Sexual and Gender Diverse Communities for Population-Based Research in Cancer Prevention and Control: Methods for Online Survey Data Collection and Management
Source: Front Oncol. 2022 Jun 8;12:841951. doi: 10.3389/fonc.2022.841951 (PMC9213655; doi:10.3389/fonc.2022.841951)
Supplement: Supplementary Appendix 1A — Eligibility survey in English. [file DataSheet_1.pdf]

CACTII-SGM Eligibility Survey

Yes, I want to help improve cancer screening services for the LGBTQI community in New Mexico!

Status

◯ Interested/ Elegible
◯ Survey sent - Complete
◯ Archive

Welcome to the CACTII - SGM study!

Thank you for your interest in participating in this important study.

The CACTII-SGM study aims at understanding the cancer screening behaviors among sexual and gender minority communities in New Mexico, that includes individuals identifying as lesbian, gay, bisexual, transgender, queer, and/or two-spirit. Findings from this survey will enable us to understand how best to promote cancer screening in this population.
Prior to participating in this study, we need to determine your eligibility.

If you need a survey in Spanish, click here https://ctsctrials.health.unm.edu/redcap/surveys/?s=94EYN7KAH9

The University of New Mexico's Human Research Protections Office has approved this study. [HRRC ID 20-393]

---

1. How did you hear about this study?
(Check all that apply)

☐ Saw a post on social media (Facebook or Twitter) Google/Gmail
☐ Received an email about the study
☐ Received a flyer in the mail
☐ A family member/ friend/ neighbor/ colleague told me about this study
☐ Other (Please specify)

Other (Please specify)

____

---

2. Were you born in the years between 1940 and 1999?

◯ Yes ◯ No

---

3. Do you identify as a lesbian, gay, bisexual, transgender, queer, or two spirit?

◯ Yes ◯ No

---

4. Are you a resident of New Mexico?

◯ Yes ◯ No

You are EligibleIf you are still interested in participating please select an option below.

The next step of this study is to answer a survey that contains questions about your body and health behaviors as they relate to cancer screening. We are hoping to create a comprehensive understanding of our LGBTQ+ communities' health as it relates to cancer prevention.

We estimate this survey to take around 15-20 minutes to complete.

Upon completion of the survey, you will be eligible to receive a $20 merchandise card as compensation for your time.

Please answer the following questions so we can send you the questionnaire.

5. Would you like to participate in the study? ◯ Yes ◯ No

6. What is your preferred language to answer the survey? ◯ English ◯ Spanish

7. How would you like to receive the survey? ◯ Email ◯ Postal mail

Please provide an email address to receive the survey ______________________

9. Please provide a postal mail address to receive the survey

Street and house number or PO Box: ______________________

City ______________________

Zip code: ______________________

Ineligible Unfortunately you are not able to participate in this research study.

Please let us know if you have any questions or suggestions for us. You can reach the study coordinator at CACTI-SGM@Salud.unm.edu / (505)925-0619

We thank you in advance for promoting this important work that will help our communities understand how the experience of being LGBTQ+ is related to cancer prevention.

Please find below a list of websites, organizations, and hotlines that may be helpful in promoting LGBTQ people's health, safety, and wellbeing.

• Find free HIV testing in your area through the Centers for Disease Control's GetTested program:https://gettested.cdc.gov/

• Find an LGBTQ+-friendly doctor through GLMA: Health Professionals Advancing LGBT Equality: https://glmaimpak.networkats.com/members_online_new/members/dir_provider.asp

Albuquerque Area Resources:

• Transgender Resource Center of New Mexico: https://tgrcnm.org/; (505) 200-9086

• Talk with someone 24/7 if you are in crisis or thinking of suicide: National Suicide Prevention Lifeline: 1-800-273-8255

• AGORA UNM Crisis Center: http://www.agoracares.org/; (505) 277-3013

• Talk with someone 24/7 if you need support related to being a survivor of sexual assault: National Sexual Assault Hotline: 1-800-656-4673

• Albuquerque SANE Collaborative: https://abqsane.org/; (505) 884-SANE

PLEASE HIT SUBMIT
