# Supplementary material for: Reaching the “Hard-to-Reach” Sexual and Gender Diverse Communities for Population-Based Research in Cancer Prevention and Control: Methods for Online Survey Data Collection and Management
Source: Front Oncol. 2022 Jun 8;12:841951. doi: 10.3389/fonc.2022.841951 (PMC9213655; doi:10.3389/fonc.2022.841951)
Supplement: Supplementary Appendix 1B — Eligibility survey in Spanish. [file DataSheet_2.pdf]

CACTII-SGM Survey

Welcome to the CACTII - SGM Study! Thank you for your interest in participating in this important study.

The CACTII-SGM study aims at understanding the cancer screening behaviors among sexual and gender minority communities in New Mexico, that includes individuals identifying as lesbian, gay, bisexual, transgender, queer, and/or two-spirit. To do that, we ask many questions. While these questions may not use perfectly appropriate language for LGBTQ+ communities, we use these questions so that we can make comparisons to the overall US population.

Please let us know, at the end of the survey, if you have suggestions for us to improve this survey.

We estimate this survey to take around 15-20 minutes to complete. We recommend that you complete the survey in one sitting.

At the end of the survey –

You may provide an email/ mailing address, where the study coordinator can send you a token of our appreciation ($20 merchandise card) You may also be asked to volunteer to participate in a focus group to talk more in depth about some of the questions. We will be conducting this focus group via telephone or zoom at a future date and ask that you let us know if you are interested in participating. Findings from this survey will enable us to understand how best to promote cancer screening in the NM's SGM community.

The University of New Mexico's Human Research Protections Office has approved this study. (HRRC study number 20-393)

Prior to participating in this study, we need to provide you more information about the study details and ensure that you have the opportunity to ask us any questions you may have regarding the study.

What are the key reasons you might choose to volunteer for this study?

Although you may personally not benefit from participating in the research study, your responses may help us understand more about the experiences of New Mexico's Sexual and Gender Minority (NM – SGM) communities.

Findings from this survey will enable us to understand how best to promote cancer screening in the NM's SGM community.

What are the key reasons you might choose not to volunteer for this study?

The survey will take approximately 15-20 minutes of your time and your participation in this study is voluntary. You may chose not to fill in the survey at any point.

Although we have tried to minimize this, some questions in the survey may make you upset or feel uncomfortable and you may choose not to answer them. If some questions do upset you, we have some resources at the University of New Mexico's LGBTQ Resource center (http://lgbtqrc.unm.edu/) or the Transgender Resource Center (https://tgrcnm.org/) that may help.

Some additional considerations regarding your participation:

You will be paid $20 after receiving a complete survey using the email/postal mailing address that you provide us. Once the distribution of the merchandise card is complete, we will delete all email addresses and postal addresses.

Your response to the survey is anonymous, which means no individual identifiable information will appear on research documents, in presentations or publications. Individual data collected through this survey will be aggregated which will be used for research reporting.

Please be aware, while we make every effort to safeguard your data once received on our servers via REDCap, given the nature of online surveys, as with anything involving the Internet, we can never guarantee the confidentiality of the data while being transmitted to us.

If you have questions about the study, please feel free to ask; my contact information is given below. If you have questions regarding your legal rights as a research subject, you may call the UNM Human Research Protections Office at (505) 272-1129.

Contact information for the Primary Investigator for this study

Dr. Prajakta Adsul, MBBS, MPH, PhD
Assistant Professor, Department of Internal Medicine,
University of New Mexico Comprehensive Cancer Center
Phone: 505-272-7351
Email: padsul@salud.unm.edu

Contact information for the study coordinator

Ms. Karen Quezada,
Study Coordinator,
University of New Mexico Comprehensive Cancer Center
Phone: 505-925-0619
Email: KaQuezada@salud.unm.edu

Contact information for The Study

Email: CACTI-SGM@Salud.unm.edu

Thank you in advance for your assistance with this important project.

To ensure your responses are included, please submit your survey by February 28th, 2021

By clicking on the "Next Page" button below, you will be agreeing to participate in the study described above.

Sincerely,

Dr. Prajakta Adsul, MBBS, MPH, PhD
Assistant Professor, Department of Internal Medicine,
University of New Mexico Comprehensive Cancer Center
Phone: 505-272-7351
Email: padsul@salud.unm.edu

Let's begin by asking a few questions about your gender identity and your sexual orientation, where you live, and other categories that may describe you.

What is your current gender identity?
(Check all that apply.)

☐ Agender
☐ Cisgender man
☐ Cisgender woman
☐ Genderqueer
☐ Man
☐ Non-binary
☐ Questioning
☐ Transgender man
☐ Transgender woman
☐ Two-spirit
☐ Woman
☐ Another gender identity (please specify)

Another gender identity (Please specify) ______________________________

What was the sex assigned to you at birth, for example on your original birth certificate?

○ Female ○ Male ○ Intersex

What is your current sexual orientation?
(Check all that apply.)

☐ Asexual
☐ Bisexual
☐ Gay
☐ Lesbian
☐ Pansexual
☐ Queer
☐ Questioning
☐ Same-gender loving
☐ Straight/Heterosexual
☐ Two-spirit
☐ Another sexual orientation (please specify)

Another sexual orientation (please specify) ______________________________

Which categories describe you?

◯ American Indian or Alaska Native (For example: Aztec, Blackfeet tribe, Mayan, Navajo Nation, Native Village of Barrow Inupiat Traditional Government, Nome Eskimo Community, etc.)
◯ Asian (For example: Asian Indian, Chinese, Filipino, Japanese, Korean, Vietnamese, etc)
◯ Black, African American or African (For example: African American, Ethiopian, Haitian, Jamaican, Nigerian, Somali, etc.)
◯ Hispanic, Latino or Spanish (For example: Colombian, Cuban, Dominican, Mexican or Mexican American, Puerto Rican, Salvadoran, etc.)
◯ Middle Eastern or North African (For example: Algerian, Egyptian, Iranian, Lebanese, Moroccan, Syrian, etc.)
◯ Native Hawaiian or other Pacific Islander (For example: Chamorro, Fijian, Marshallese, Native Hawaiian, Tongan, etc.)
◯ White (For example: English, European, French, German, Irish, Italian, Polish, etc.)
◯ None of these fully describe me/ More than one of these describe me. (Please specify)

None of these fully describe me/ More than one of these describe me. (Please specify) ______________________________

Which additional categories describe you? (Check all that apply)

☐ American Indian
☐ Alaska Native
☐ Central or South American Indian
☐ None of these fully describe me/ More than one of these describe me (Please specify)

None of these fully describe me/ More than one of these describe me (Please specify) ______________________________

Which of these groups contains your age?

◯ 21-25 years
◯ 26-30 years
◯ 31-35 years
◯ 36-40 years
◯ 41-45 years
◯ 46-50 years
◯ 51-55 years
◯ 56-60 years
◯ 61-65 years
◯ 66-70 years
◯ 71-75 years
◯ 76-80 years

What is your current height in feet and inches? If you don't know, please give your best estimate

---

Feet ______________________________
(only numerical values)

Inches ______________________________
(only numerical values)

What is your current weight in pounds (lbs)? If you don't know, please give your best estimate

---

lbs ______________________________
(only numerical values)

What is your best estimate (in US dollars) of your household earnings before taxes and deductions from ALL sources (including jobs, businesses, welfare, child support, disability, social security, etc.) in the 2018 tax year?

◯ $0
◯ $1 - $10,000
◯ $10,000 - $20,000
◯ $20,000 - $30,000
◯ $30,000 - $40,000
◯ $40,000 - $50,000
◯ $50,000 - $60,000
◯ $60,000 - $70,000
◯ $70,000 - $80,000
◯ $80,000 - $90,000
◯ $90,000 - $100,000
◯ $100,000+

What is your highest education level completed?

◯ No schooling
◯ Nursery school to high school, no diploma
◯ High school graduate or equivalent (e.g., GED)
◯ Trade/Technical/Vocational training
◯ Some college
◯ 2-year college degree
◯ 4-year college degree
◯ Master's degree
◯ Doctoral degree
◯ Professional degree (e.g., M.D., J.D., M.B.A.)

Do you currently work one or more paid jobs? ◯ Yes ◯ No

Which of the following describes your current occupation (Check all that apply)

☐ Employed, working 40 or more hours per week
☐ Employed, working 1-39 hours per week
☐ Temporarily employed
☐ Self-employed
☐ Not employed, looking for work
☐ Not employed, not looking for work
☐ Homemaker
☐ Student (Full time)
☐ Student (Half time)
☐ Disabled, not able to work
☐ Retired

What is your ZIP code? (This is the 5-digit code that helps direct U.S. Mail to you.) ______________________

How did you hear about this study (Check all that apply)

☐ Saw a post on social media (Facebook, Twitter) or Google/Gmail
☐ Received an email about the study
☐ Received a flyer in the mail
☐ A family member/ friend/ neighbor/ colleague told me about this study
☐ Other (Please specify)

Other (Please specify) ______________________

The next set of questions mentions body organs. We are asking these questions to get a comprehensive look at your health. We know that people refer to their organs differently, and we have tried to use the medical terms as well as commonly used non-medical terms.

We know that this will not accurately reflect all the diversity of our communities, but we hope it gets us closer to some critical health understanding.

To understand your health and customize this survey for you, we need to know what organs you were born with. People have a wide range of language or terms for their physical anatomy (not all of which are listed here).

Which of the following organs do you have now? (Check all that apply.)

☐ Breasts or breast tissue
☐ Cervix (you likely have/had this if you were assigned female sex at birth)
☐ Ovaries
☐ Uterus/Womb
☐ Vagina/Frontal genital opening
☐ Penis/Phallus (not including a prosthetic)
☐ Prostate (you likely have/had this if you were assigned male sex at birth)
☐ Testicles

This section of the survey is meant to give us a sense of your physical health.

Have you EVER tried cigarette smoking, even one or two puffs? ◯ Yes ◯ No

Have you smoked at least 100 cigarettes in YOUR ENTIRE LIFE? ◯ Yes ◯ No

In the past MONTH, have you used any tobacco or nicotine products other than cigarettes? (Check all that apply)

☐ Blunt (with another substance)
☐ Blunt (without any other substance)
☐ Bidi
☐ Chewing tobacco ("chew")
☐ Other cigars with tobacco inside (e.g. cigarillos, little cigars, bidis)
☐ Other cigars with another substance (e.g. cigarillos, little cigars, bidis)
☐ Dip
☐ E-cigarette or vape device with nicotine
☐ E-cigarette or vape device without nicotine
☐ Nicotine replacement products
☐ Snuff
☐ Snus
☐ Other tobacco or nicotine containing product (please specify)
☐ I have never used any tobacco product other than cigarettes
☐ I have never used any tobacco- or nicotine-containing products

Other tobacco or nicotine containing product (please specify) __________________________

How often did you have a drink containing alcohol in the PAST YEAR?

◯ Never
◯ Monthly or less
◯ 2-4 times a month
◯ 2-3 times a week
◯ 4 or more times a week

How many drinks containing alcohol did you have on a typical day when you were drinking in the PAST YEAR?

◯ 1 or 2
◯ 3 or 4
◯ 5 or 6
◯ 7-9
◯ 10 or more

How long has it been since you had 5 or more drinks containing alcohol on one occasion?

◯ Within past 30 days
◯ More than 30 days ago but within the past 12 months
◯ More than 12 months ago
◯ Never had 5 or more drinks on one occasion

Survey 25% Complete

Have you been diagnosed by a health care provider to have cancer

◯ Yes ◯ No

With what type(s) of cancer have you been diagnosed (Check all that apply)

☐ Anal
☐ Breast
☐ Cervix
☐ Colon
☐ Kidney
☐ Lung
☐ Leukemia/Lymphoma
☐ Ovary
☐ Pancreas
☐ Prostate
☐ Skin (melanoma)
☐ Skin (non-melanoma)
☐ Uterus
☐ Other (please specify)

Other (please specify)

____

In order to customize the rest of this questionnaire, please select the term you would like us to use to describe your vagina/frontal genital opening.

Please use the term

◯ vagina
◯ frontal genital opening

This section asks about cancer screening.
Please do your best to answer every question, but you may skip questions that feel too uncomfortable to answer. Completing the whole questionnaire means we have more power to advance LGBTQ+ health. Thank you for making a difference!

Have you EVER had a Pap smear or Pap test? (A Pap smear or Pap test is a routine test in which a health care provider places an instrument inside the [preferred_term], examines the cervix, and takes a few cells from the cervix with a small stick or brush to look for abnormal or cancer cells.)

◯ Yes
◯ No
◯ I don't know

In the PAST 3 YEARS, have you had a Pap smear or Pap test? (A Pap smear or Pap test is a routine test in which a health care provider places an instrument inside the [preferred_term], examines the cervix, and takes a few cells from the cervix with a small stick or brush to look for abnormal or cancer cells.)

◯ Yes
◯ No
◯ I don't know

How long has it been since your last Pap smear or Pap test?

◯ A year ago or less
◯ More than 1 year but not more than 2 years ago
◯ More than 2 years but not more than 3 years ago
◯ More than 3 years but not more than 5 years ago
◯ Over 5 years ago
◯ I don't know

What is the most important reason you have NEVER had a Pap test?

◯ I do not have a reason or I never thought about it
◯ I did not know I needed this type of test
◯ I did not know this test existed
◯ My health care provider told me I did not need it
◯ I have not had any problems
◯ I put it off or I did not get around to it
◯ It was too expensive
◯ I have no insurance
◯ It was too painful, unpleasant, or embarrassing
◯ I do not have a cervix or I have had a hysterectomy
◯ I do not have a provider
◯ I had an HPV vaccine
◯ No one I know has had one
◯ I don't know
◯ Other (Please specify)

Other (Please specify)

____

What is the most important reason you have NOT had a Pap test in the LAST 3 YEARS?

◯ I do not have a reason or I never thought about it
◯ I did not know I needed this type of test
◯ I did not know this test existed
◯ My health care provider told me I did not need it
◯ I have not had any problems
◯ I put it off or I did not get around to it
◯ It was too expensive
◯ I have no insurance
◯ It was too painful, unpleasant, or embarrassing
◯ I do not have a cervix or I have had a hysterectomy
◯ I do not have a provider
◯ I had an HPV vaccine
◯ No one I know has had one
◯ I don't know
◯ Other (Please specify)

Other (Please specify) __________________________________

Have you EVER had a Pap test where the results were NOT normal?

◯ Yes
◯ No
◯ I don't know

HPV - Human Papillomavirus

An Human PapillomaVirus (HPV) test is sometimes added to the Pap test for cervical cancer screening. Have you EVER had an HPV test along with your cervical Pap test?

◯ Yes
◯ No
◯ I don't know

Did you have an HPV test with a Pap test in the PAST 5 YEARS ?

◯ Yes
◯ No
◯ I don't know

Did you have an HPV test without a Pap test in the PAST 5 YEARS

◯ Yes
◯ No
◯ I don't know

Have you EVER had a HPV test where the results were positive (meaning you were positive for HPV)?

◯ Yes
◯ No
◯ I don't know

Have you EVER had a mammogram? A mammogram is when breast/chest tissue is squeezed between two firm surfaces to obtain X-rays/pictures of the breast/chest tissue.

◯ Yes
◯ No
◯ I don't know

What is the most important reason you have NEVER had a mammogram?

◯ I do not have a reason or I never thought about it
◯ I did not know I needed this type of test
◯ I did not know this test existed
◯ My health care provider told me I did not need it
◯ I have not had any problems
◯ I put it off or I did not get around to it
◯ It was too expensive
◯ I have no insurance
◯ It was too painful, unpleasant, or embarrassing
◯ I do not have a cervix or I have had a hysterectomy
◯ I do not have a provider
◯ No one I know has had one
◯ I don't know
◯ Other (Please specify)

Other (Please specify)

____

In the PAST 2 YEARS, have you had a mammogram? A mammogram is when breast/chest tissue is squeezed between two firm surfaces to obtain X-rays/pictures of the breast/chest tissue.

◯ Yes
◯ No
◯ I don't know

Have you had a mammogram in the PAST 2 YEARS where the results were NOT normal?

◯ Yes
◯ No
◯ I don't know

How long has it been since your last mammogram?

◯ A year ago or less
◯ More than 1 year but not more than 2 years ago
◯ More than 2 years but not more than 3 years ago
◯ More than 3 years but not more than 5 years ago
◯ Over 5 years ago
◯ I don't know

What is the most important reason you have NOT had a mammogram test in the LAST 2 YEARS?

◯ I do not have a reason or I never thought about it
◯ I did not know I needed this type of test
◯ I did not know this test existed
◯ My health care provider told me I did not need it
◯ I have not had any problems
◯ I put it off or I did not get around to it
◯ It was too expensive
◯ I have no insurance
◯ It was too painful, unpleasant, or embarrassing
◯ I do not have a cervix or I have had a hysterectomy
◯ I do not have a provider
◯ No one I know has had one
◯ I don't know
◯ Other (Please specify)

Other (Please specify) __________________________________

Have you EVER had a mammogram where the results were NOT normal?

◯ Yes
◯ No
◯ I don't know

Have you EVER had a PSA test? A Prostate-Specific Antigen (PSA) test is a blood test to detect prostate cancer.

◯ Yes
◯ No
◯ I don't know

What is the most important reason you have NEVER had a PSA test?

◯ I do not have a reason or I never thought about it
◯ The test is not recommended for me
◯ I did not know I needed this type of test
◯ I did not know this test existed
◯ My health care provider told me I did not need it
◯ I have not had any problems
◯ I put it off or I did not get around to it
◯ It was too expensive
◯ I have no insurance
◯ It was too painful, unpleasant, or embarrassing
◯ I do not have a provider
◯ No one I know has had one
◯ I don't know
◯ Other (Please specify)

Other (Please specify) ________________________________

Who first suggested the PSA test?

◯ I did
◯ My health care provider did
◯ Someone else
◯ I don't know

Did a doctor or health care provider EVER talk with you about the advantages of the PSA test?

◯ Yes
◯ No
◯ I don't know

In the PAST 12 MONTHS, have you had a PSA test?

◯ Yes
◯ No
◯ I don't know

Have you had a PSA test in the PAST 12 MONTHS where the results were NOT normal?

◯ Yes
◯ No
◯ I don't know

Survey 50% Complete
Colon or rectal cancer tests include blood stool tests, colonoscopy, and sigmoidoscopy.

A blood stool test or occult blood test, also known as the fecal immunochemical (FIT) test, determines whether you have blood in your stool or bowel movement. These tests can be done at home using a kit. You use a stick or brush to obtain a small amount of stool at home and send it back to the doctor or lab.

A sigmoidoscopy and colonoscopy are exams in which a tube is inserted in the rectum to view the colon for signs of cancer or other health problems. Before a sigmoidoscopy or colonoscopy, you are asked to take a medication that causes diarrhea.

For a sigmoidoscopy, the doctor or another health care provider checks only part of the colon and you are fully awake.
For a colonoscopy, the doctor or another health care provider checks the entire colon, and you are given medication through a needle in your arm to make you sleepy, and told to have someone drive you home.

Have you EVER had any of these tests for colon or rectal cancer? (Check all that apply.)

☐ None of these
☐ Blood stool test (FIT test)
☐ Sigmoidoscopy
☐ Colonoscopy

What is the most important reason you have NEVER had any of these tests to test for colon or rectal cancer?

◯ I do not have a reason or I never thought about it
◯ I did not know I needed this type of test
◯ I did not know this test existed
◯ My health care provider told me I did not need it
◯ I have not had any problems
◯ I put it off or I did not get around to it
◯ It was too expensive
◯ I have no insurance
◯ It was too painful, unpleasant, or embarrassing
◯ I do not have a provider
◯ I am under the age of 50
◯ No one I know has had one
◯ I don't know
◯ Other (Please specify)

Other (Please specify)

____

How long has it been since your last blood stool test (FIT test)?

◯ A year ago or less
◯ More than 1 year but not more than 2 years ago
◯ More than 2 years but not more than 3 years ago
◯ More than 3 years but not more than 5 years ago
◯ More than 5 years but not more than 10 years ago
◯ Over 10 years ago
◯ I don't know

What is the most important reason you have NOT had a blood stool test (FIT test) in the LAST 1 YEAR?

◯ I do not have a reason or I never thought about it
◯ I did not know I needed this type of test
◯ I did not know this test existed
◯ My health care provider told me I did not need it
◯ I have not had any problems
◯ I put it off or I did not get around to it
◯ It was too expensive
◯ I have no insurance
◯ It was too painful, unpleasant, or embarrassing
◯ I do not have a provider
◯ I am under the age of 50
◯ I am over the age of 75
◯ No one I know has had one
◯ I don't know
◯ Other (Please specify)

Other (Please specify) ______________________________

Have you EVER had a blood stool test (FIT) where the results were NOT normal?

◯ Yes
◯ No
◯ I don't know

How long has it been since your last sigmoidoscopy

◯ A year ago or less
◯ More than 1 year but not more than 2 years ago
◯ More than 2 years but not more than 3 years ago
◯ More than 3 years but not more than 5 years ago
◯ More than 5 years but not more than 10 years ago
◯ Over 10 years ago
◯ I don't know

What is the most important reason you have NOT had a sigmoidoscopy in the LAST 10 YEARS?

◯ I do not have a reason or I never thought about it
◯ I did not know I needed this type of test
◯ I did not know this test existed
◯ My health care provider told me I did not need it
◯ I have not had any problems
◯ I put it off or I did not get around to it
◯ It was too expensive
◯ I have no insurance
◯ It was too painful, unpleasant, or embarrassing
◯ I do not have a provider
◯ No one I know has had one
◯ I don't know
◯ Other (Please specify)

Other (Please specify) __________________________________

Have you EVER had a sigmoidoscopy where the results were NOT normal?

◯ Yes
◯ No
◯ I don't know

How long has it been since your last colonoscopy?

◯ A year ago or less
◯ More than 1 year but not more than 2 years ago
◯ More than 2 years but not more than 3 years ago
◯ More than 3 years but not more than 5 years ago
◯ More than 5 years but not more than 10 years ago
◯ Over 10 years ago
◯ I don't know

What is the most important reason you have NOT had a colonoscopy test in the LAST 10 YEARS?

◯ I do not have a reason or I never thought about it
◯ I did not know I needed this type of test
◯ I did not know this test existed
◯ My health care provider told me I did not need it
◯ I have not had any problems
◯ I put it off or I did not get around to it
◯ It was too expensive
◯ I have no insurance
◯ It was too painful, unpleasant, or embarrassing
◯ I do not have a provider
◯ No one I know has had one
◯ I don't know
◯ Other (Please specify)

Other (Please specify) __________________________________

Have you EVER had a colonoscopy where the results were NOT normal?

◯ Yes
◯ No
◯ I don't know

Have you EVER had any of the following tests as an evaluation for anal or rectal cancer? (Check all that apply.)

☐ Digital anal rectal exam (an examination where a doctor or health care provider inserts their finger into your anus (butt)
☐ Anal HPV test (a routine test with a swab that tests for human papillomavirus, HPV)
☐ Anal Pap smear (a routine test in which a health care provider takes a few cells from the anus (butt) using a swab to look for abnormal or cancer cells)
☐ High-Resolution Anoscopy (HRA) (an exam with a microscope of the rectum and anus/butt)
☐ I don't know
☐ None of these

Have you EVER had an anal HPV test where the results were NOT normal?

◯ Yes
◯ No
◯ I don't know

Have you EVER had an anal Pap test where the results were NOT normal?

◯ Yes
◯ No
◯ I don't know

Have you EVER had a screening low-dose Computerized Tomography (CT or CAT scan) to screen for lung cancer?

◯ Yes
◯ No
◯ I don't know

Have you EVER had a low-dose CT scan where the results were NOT normal

◯ Yes
◯ No
◯ I don't know

How long has it been since your last low-dose CT scan?

◯ A year ago or less
◯ More than 1 year but not more than 2 years ago
◯ More than 2 years but not more than 3 years ago
◯ More than 3 years but not more than 5 years ago
◯ Over 5 years ago
◯ I don't know

What is the most important reason you have NEVER had a CT test?

◯ I do not have a reason or I never thought about it
◯ I did not know I needed this type of test
◯ My health care provider told me I did not need it
◯ I have not had any problems
◯ I put it off or I did not get around to it
◯ It was too expensive
◯ I have no insurance
◯ I do not have a provider
◯ I don't know
◯ Other (Please specify)

Other (Please specify) ________________________________

Survey 75% Complete

Have you EVER received an HPV shot or vaccine? HPV stands for human papillomavirus. The vaccines are sometimes called CERVARIX® or GARDASIL®. The HPV vaccine is given as a three-dose series routinely to people from age 9-45. It was released in 2006.

◯ Yes
◯ No
◯ I don't know

What is the most important reason you NEVER received the HPV vaccine?

◯ I do not have a reason or I never thought about it
◯ I did not know I needed this vaccine
◯ I did not know this test existed
◯ Doctor or another health care provider refused to give it to me when I asked for it
◯ My health care provider told me I did not need it
◯ I put it off or I did not get around to it
◯ It was too expensive
◯ I have no insurance
◯ It was too painful, unpleasant, or embarrassing
◯ I do not have a cervix or I have had a hysterectomy
◯ I do not have a provider
◯ No one I know has had one
◯ I don't know
◯ Other (Please specify)

Other (Please specify)

____

How many HPV vaccine shots did you have?

◯ One
◯ Two
◯ Three
◯ I don't know

This last section of the survey is meant to give us a sense of your access to healthcare.

Many of these questions are standard questions routinely asked in national health surveys. Your honest answers will help us as we study LGBTQ+ health in order to improve the health and well-being of our communities in New Mexico.

Are you currently covered by any health insurance or health coverage plan?

◯ Yes
◯ No
◯ I don't know

Are you currently covered by any of the following types of health insurance or health coverage plans? (If you have more than one insurance/coverage plans, please select your primary insurance/coverage plan.)

◯ Insurance through my current or former employer or union
◯ Insurance through someone else's current or former employer or union
◯ Insurance purchased through HealthCare.gov or another health insurance marketplace (sometimes called "Obamacare" or the "Affordable Care Act")
◯ Insurance purchased directly from an insurance company
◯ Medicare (for people 65 and older or people with certain disabilities)
◯ Medicaid (government-assistance plan for those with low incomes or a disability)
◯ TRICARE or other military health care
◯ Veteran's Affair (VA)
◯ Indian Health Service
◯ Other (Please specify)

Other (Please specify) __________________________________

Were you uninsured for any time during the previous 12 months?

◯ Yes
◯ No
◯ I don't know

Is there a place that you USUALLY go to when you need routine or preventive care, such as a physical examination or check-up?

◯ Yes
◯ No
◯ I don't know

During the past 12 months, did you have trouble finding a general doctor or health care provider who would see you?

◯ Yes
◯ No
◯ I haven't tried to see a doctor or health care provider in the past 12 months
◯ I don't know

If you have had trouble finding a general doctor or health care provider in the past 12 months, what is the reason?

◯ Closest provider was too far away
◯ I did not think I needed it
◯ I am worried about discrimination
◯ It is too expensive
◯ I do not have reliable transportation
◯ I do not trust healthcare system
◯ I have other priorities
◯ I do not have health insurance
◯ I do not trust local providers
◯ Other (please specify)

Other (please specify)

____

A primary care provider is a health care provider who takes care of your overall general health and may coordinate your care with other medical specialists. Do you have a primary care provider?

◯ Yes
◯ No
◯ I don't know

Have you seen your primary care provider in the PAST 12 MONTHS?

◯ Yes
◯ No
◯ I don't know

In the PAST 12 MONTHS, were you delayed in getting medical care, tests, or treatments that you or a health care provider believed necessary?

◯ Yes
◯ No
◯ Not applicable

In the PAST 12 MONTHS, were you unable to obtain medical care, tests, or treatments that you or a health care provider believed necessary?

◯ Yes
◯ No
◯ Not applicable

In the PAST 12 MONTHS, have you been denied or given lower quality medical care?

◯ Yes
◯ No
◯ Not applicable

Do you think you were targeted for this discrimination in a medical setting in the PAST 12 months due to your ... (Check all that apply)

- ☐ Ability/disability status
- ☐ Age
- ☐ Body size, weight, or shape
- ☐ Gender expression
- ☐ Gender identity
- ☐ Race and/or ethnicity
- ☐ Sexual orientation
- ☐ Something else (please specify)
- ☐ None of the above

Something else (please specify) ______________________________

Is there anything else you would like to share with us about your health or well-being?

Survey 90% Complete

You are almost done with the survey.

Please read below and indicate your interest.
This survey is part of a larger research project that is trying to understand how best to promote the uptake of cervical cancer screening among lesbian, bisexual, queer, cisgender women, transgender men, and gender non-conforming individuals with a cervix. We are hoping to talk to individuals in the community to better understand some of the barriers to getting care and issues that might prevent people in these groups from accessing cervical cancer screening.

This part of the study will consist of participating in focus group discussion with a few other participants and an interviewer. Participation in this foocus group will be anonymous and require you to answering questions posted by the interviewer and interact with other participants

Would you be willing to talk to us about your experiences in seeking cancer screening services?

◯ Yes ◯ No

Please provide an email or phone number on which we can reach you. (We will not share this information with anyone outside the study team and this information will not be linked to the responses you provided above)

Thank you for your participation!

How would you like us to send you this merchandise card?

◯ Online merchandise card code ◯ Postal mail

Please provide an email where we can send your merchandise card. (We will not share this information with anyone outside the study team and this information will not be linked to the responses you provided above)

____

Please provide mailing address where we can send your merchandise card. (We will not share this information with anyone outside the study team and this information will not be linked to the responses you provided above)

Street address/PO BOX __________________________________

City __________________________________

State __________________________________

Zip __________________________________

You are done with the survey.

We are committed to communicating findings from the study back to our community.

As mentioned earlier, your response to the survey is anonymous, which means no individual identifiable information will appear on research documents, in presentations or publications.

Individual data collected through this survey will be aggregated which will be used for research reporting.

If you are interested in learning more about our study and the study finding, please answer the following questions.

Would you like to hear from us regarding the study findings? ◯ Yes ◯ No

How would you like us to send you future communications? ◯ Email ◯ Postal mail

Please provide an email where we can send you future communications. (We will not share this information with anyone outside the study team and this information will not be linked to the responses you provided above)

____

Please provide mailing address where we can send you future communications. (We will not share this information with anyone outside the study team and this information will not be linked to the responses you provided above)

Street address/PO BOX __________________________________

City __________________________________

State __________________________________

Zip Code __________________________________

Survey 100% Complete
Thank you for making a difference!

In addition to our commitment to communicating findings from the study back to our community in the future, we also want to connect our participants with some resources that may be helpful to them now. Please find below a list of websites, organizations, and hotlines that may be helpful in promoting LGBTQ people's health, safety, and wellbeing.

• Find free HIV testing in your area through the Centers for Disease Control's GetTested program:https://gettested.cdc.gov/

• Find an LGBTQ+-friendly doctor through GLMA: Health Professionals Advancing LGBT Equality: https://glmaimpak.networkats.com/members_online_new/members/dir_provider.asp

Albuquerque Area Resources:
• Transgender Resource Center of New Mexico: https://tgrcnm.org/; (505) 200-9086
• Talk with someone 24/7 if you are in crisis or thinking of suicide: National Suicide Prevention Lifeline: 1-800-273-8255

• AGORA UNM Crisis Center: http://www.agoracares.org/; (505) 277-3013
• Talk with someone 24/7 if you need support related to being a survivor of sexual assault: National Sexual Assault Hotline: 1-800-656-4673

• Albuquerque SANE Collaborative:https://abqsane.org/; (505) 884-SANE

Thank you again for completing the survey.

We deeply appreciate for your time, your interest in this study and your investment in research that will help our communities understand how the experience of being LGBTQ+ is related to all aspects of health and life.

PLEASE HIT SUBMIT

Please write any comments below

CLICK "SUBMIT" TO COMPLETE SURVEY
