# Supplementary material for: Reaching the “Hard-to-Reach” Sexual and Gender Diverse Communities for Population-Based Research in Cancer Prevention and Control: Methods for Online Survey Data Collection and Management
Source: Front Oncol. 2022 Jun 8;12:841951. doi: 10.3389/fonc.2022.841951 (PMC9213655; doi:10.3389/fonc.2022.841951)
Supplement: Supplementary Appendix 2A — Cancer prevention practices survey in English. [file DataSheet_3.pdf]

CACTII - SGM Survey

¡Bienvenidxs al estudio CACTII-SGM! Gracias por su interés en participar en este estudio importante.

El estudio CACTII-SGM tiene como objetivo comprender los comportamientos de prevención del cáncer entre las comunidades de minorías sexuales y de género en Nuevo México, que incluye a personas que se identifican como lesbianas, gays, bisexuales, transgénero, queer o de dos espíritus. Para poder analizar esos comportamientos, es necesario hacer muchas preguntas. Estas preguntas pueden usar lenguaje que no es perfectamente apropiado para las comunidades LGBTQ+ sin embargo, las usamos para poder hacer comparaciones con la población general del EE. UU.

Al final de la encuesta, por favor háganos saber si tiene sugerencias para mejorar esta encuesta.

Estimamos que esta encuesta tomará entre 15 a 20 minutos en completarse. Le recomendamos que complete la encuesta en una sola sesión.

Al final de la encuesta

Puede proporcionar su dirección de correo electrónico/postal, para que el coordinador del estudio le envié un agradecimiento (tarjeta de mercancía de $20) También puede anotarse como voluntarix para participar en un grupo de enfoque, donde hablaremos en mas detalle sobre algunas de las preguntas. Llevaremos a cabo este grupo por llamada telefónica o por Zoom, algún momento en el futuro, y le pediremos que nos informe si está interesadx en participar. Los resultados de esta encuesta nos permitirán comprender cuál es la mejor manera de promover la detección del cáncer en la comunidad SGM de NM.

La Oficina de Protección de la Investigación Humana de la Universidad de Nuevo México aprobó este estudio. (Numero de Estudio HRRC 20-393)

Antes de participar en este estudio, debemos brindarle más información sobre los detalles del estudio y asegurarnos de que tenga la oportunidad de hacernos cualquier pregunta que pueda tener con respecto al estudio.

¿Cuáles son las razones principales para participar en este estudio?

Aunque es posible que no se beneficie personalmente al participar en este estudio, sus respuestas pueden ayudarnos a comprender más sobre las experiencias de las comunidades de minorías sexuales y de género (SGM) de Nuevo México.

Los resultados de esta encuesta nos permitirá comprender cuál es la mejor manera de promover la detección del cáncer en la comunidad SGM de NM.

¿Cuáles son las razones principales por las cual optaría no participar en este estudio?

La encuesta tomará aproximadamente 15 a 20 minutos de su tiempo y su participación es completamente voluntaria. Puede optar por no completar la encuesta en cualquier momento.

Aunque hemos tratado de minimizar esto, algunas de las preguntas lx pueden incomodar o molestar, y puede optar por no responder esas preguntas. Si se siente incomodx, tenemos algunos recursos en el centro de recursos LGBTQ+ de la Universidad de Nuevo México (http://lgbtqrc.unm.edu/) o el Centro de recursos transgénero (https://tgrcnm.org/) que pueden ayudar.

Algunas consideraciones adicionales con respecto a su participación:

Despues de completar la encuesta, se le pagará $20 utilizando el correo electrónico/ dirección postal que nos proporcione. Una vez que se complete la distribución de las tarjetas de mercancía, eliminaremos todas las direcciones de correo electrónico o postal.

Su participación y respuestas en la encuesta son anónimas, lo que significa que no aparecerá información de identificación individual en los documentos del estudio, presentaciones o publicaciones. Los datos individuales recopilados a través de esta encuesta se agregarán y se utilizarán para informes de investigación.

Tenga en cuenta que, aunque hacemos todo lo posible para proteger sus datos una vez recibidos en nuestros servidores a través de REDCap, dada la naturaleza de las encuestas en línea, como con cualquier cosa que involucre internet, nunca podemos garantizar la confidencialidad de los datos mientras se nos transmiten.

Si tiene preguntas sobre el estudio, no dude en preguntar; mi información de contacto se da a continuación. Si tiene preguntas sobre sus derechos legales como sujeto de investigación, puede llamar a la Oficina de Protección de Investigaciones Humanas de UNM al (505) 272-1129

Información de contacto del Investigador Principal de este estudio

Dr. Prajakta Adsul, MBBS, MPH, PhD

Profesora asistente, Departamento de Medicina Interna,
Centro Comprensivo de Cáncer de la Universidad de Nuevo Mexico
Teléfono: 505-272-7351
Correo eléctronico: padsul@salud.unm.edu

Contact information for the study coordinator

Ms. Karen Quezada,
Coordinadora del Estudio,

Centro Comprensivo de Cáncer de la Universidad de Nuevo Mexico
Teléfono: 505-925-0619
Correo eléctronico: KaQuezada@salud.unm.edu

Información de contacto para el Estudio

Correo electrónico: CACTI-SGM@Salud.unm.edu

Teléfono: (505) 925-0619

Gracias de antemano por su ayuda con este proyecto importante.

Para asegurarse que se incluyan sus respuestas, por favor complete y regrese su encuesta antes del 28 de Febrero, 2021

Al hacer clic en "Next Page" a continuación estará aceptando participar en el estudio descrito anteriormente.

Sinceramenete,
Dr. Prajakta Adsul, MBBS, MPH, PhD
Profesora asistente, Departamento de Medicina Interna, Centro Comprensivo de Cáncer de la Universidad de Nuevo Mexico
Teléfono: 505-272-7351
Correo eléctronico: padsul@salud.unm.edu

Comencemos haciendo algunas preguntas sobre usted, su identidad de género y su orientación sexual, dónde vive y otras categorías que pueden describirlx

¿Cuál es su identidad de género actual?
(Marque todo lo que corresponda)

☐ Agénero
☐ Hombre cisgénero
☐ Mujer cisgénero
☐ Género queer
☐ Hombre
☐ No-binario
☐ Cuestionándose
☐ Hombre transgénero
☐ Mujer transgénero
☐ Dos-espíritus
☐ Mujer
☐ Otra identidad de género (por favor especifique)

Otra identidad de género (por favor especifique)

____

¿Cuál fue el sexo que se le asignó al nacer, por ejemplo, en su certificado de nacimiento original?

◯ Femenino
◯ Masculino
◯ Intersexual

¿Cuál es su orientación sexual actual?
(Marque todo lo que corresponda.)

☐ Asexual
☐ Bisexual
☐ Gay
☐ Lesbiana
☐ Pansexual
☐ Queer
☐ Cuestionándose
☐ Amante del mismo género
☐ Heterosexual
☐ Dos-espíritus
☐ Otra orientación sexual (por favor especifique)

Otra orientación sexual (por favor especifique) __________________________________

¿Qué categorías te describen? (Marque todo lo que corresponda.)

◯ Indígena americano o nativx de Alaska (por ejemplo: azteca, tribu Blackfeet, maya, nación Najavo, pueblo nativo de Barrow, gobierno tradicional inupiat, comunidad esquimal Nome, etc.)
◯ Asiáticx (por ejemplo: asiáticx indix, chinx, filipinx, japonés, coreanx, vietnamita etc.)
◯ Negrx, afroamericanx o africanx (por ejemplo: afroamericanx, etíope, haitianx, jaimaicanx, nigerianx, somalí, etc.)
◯ Hispanx, latinx o españolx (por ejemplo: colombianx, cubanx, dominicanx,
◯ Medio Oriente o África del Norte (por ejemplo: argelinx, egipcix, iraní, libanés, marroquí, sirio, etc)
◯ Nativx de Hawái o de otras islas del Pacífico (por ejemplo: chamorro, fiyiano, marshalés, nativx de Hawái, tongano, etc.)
◯ Blanco (por ejemplo: inglés, europex, francés, alemánx, irlandésx, italianx, polacx
◯ Ninguno de estos me describe completamente / Más de uno de estos me describe (por favor especifique)

Ninguno de estos me describe completamente / Más de uno de estos me describe. (por favor especifique) __________________________________

¿Qué categorías adicionales te describen? (Marque todo lo que corresponda)

☐ Indix americanx
☐ nativx de Alaska
☐ Indix de América Central o Sur
☐ Ninguno de estos me describe (por favor especifique)

Ninguno de estos me describe. (Por favor cuéntenos sobre categorías adicionales que lx describan) __________________________________

¿Cuál de estos grupos tiene su edad?

◯ 21-25 años
◯ 26-30 años
◯ 31-35 años
◯ 36-40 años
◯ 41-45 años
◯ 46-50 años
◯ 51-55 años
◯ 56-60 años
◯ 61-65 años
◯ 66-70 años
◯ 71-75 años
◯ 76-80 años

Cuál es su altura actual en pies y pulgadas? Si no lo sabe, proporcione su mejor estimación

---

Pies

____
(solo valores numéricos)

Pulgadas

____
(solo valores numéricos)

¿Cuál es su peso actual en libras (lbs)? Si no lo sabe, proporcione su mejor estimación

---

lbs

____
(solo valores numéricos)

¿Cuál es su mejor estimación (en dólares estadounidenses) de los ingresos de su hogar antes de impuestos y deducciones de TODAS las fuentes (incluidos trabajos, negocios, asistencia social, manutención infantil, discapacidad, seguridad social, etc.) en el año fiscal 2020?

◯ $0
◯ $1 - $10,000
◯ $10,000 - $20,000
◯ $20,000 - $30,000
◯ $30,000 - $40,000
◯ $40,000 - $50,000
◯ $50,000 - $60,000
◯ $60,000 - $70,000
◯ $70,000 - $80,000
◯ $80,000 - $90,000
◯ $90,000 - $100,000
◯ $100,000+

¿Cuál es su nivel educativo más alto completado?

- ◯ Sin escolaridad
- ◯ Primaria hasta la preparatoria, sin diploma
- ◯ Graduadx de la preparatoria o equivalente (e.g. GED)
- ◯ Formación comercial / técnica / profesional
- ◯ Algo de colegio
- ◯ Título universitario de 2 años
- ◯ Título universitario de 4 años
- ◯ Maestría
- ◯ Doctorado
- ◯ Título profesional (e.g., M.D., J.D., M.B.A.)

¿Tiene actualmente uno o más trabajos remunerados? ◯ Sí ◯ No

¿Cuál de las siguientes describe su ocupación actual? (Marque todo lo que corresponda)

- ☐ Empleadx, trabajando 40 horas por semana
- ☐ Empleadx, trabajando 1-39 horas por semana
- ☐ Temporalmente empleadx
- ☐ Trabajando por cuenta propia
- ☐ No empleadx, buscando trabajo
- ☐ No empleadx, no buscando trabajo
- ☐ Amx de casa
- ☐ Estudiante (Tiempo completo)
- ☐ Estudiante (Medio tiempo)
- ☐ Discapacitadx, incapaz de trabajar
- ☐ Retiradx

¿Cuál es su código postal? (Este es el código de 5 dígitos que ayuda a enviarle el correo de los EE. UU.) ______________________

¿Cómo se enteró de este estudio?

- ☐ Vi una publicación en redes sociales (Facebook, Twitter) o Google/Gmail
- ☐ Recibí un correo electronico sobre el estudio
- ☐ Recibí un volante por correo
- ☐ Un familiar/ amigo/ vecino/ colega me contó sobre este estudio
- ☐ Otro (por favor especifique)

Otro (por favor especifique) ______________________

El siguiente grupo de preguntas menciona los órganos del cuerpo. Hacemos estas preguntas para obtener una visión integral de su salud. Sabemos que las personas se refieren a sus órganos de manera diferente y hemos intentado utilizar los términos médicos, y términos no médicos de uso común.

Sabemos que esto no reflejará con precisión toda la diversidad de nuestras comunidades, pero esperamos que nos acerque a una comprensión crítica de la salud.

Para comprender su salud y personalizar esta encuesta para usted, necesitamos saber con qué órganos nació. Las personas tienen una amplia gama de lenguaje o términos para su anatomía física (no todos se enumeran aquí).

¿Cuál de los siguientes órganos tienes ahora? (Marque todo lo que corresponda.)

☐ Senos o tejido mamario
☐ Cuello uterino (es probable que lo tenga/haya tenido si se le asignó sexo femenino al nacer)
☐ Ovarios
☐ Útero / matriz
☐ Vagina / apertura genital frontal
☐ Pene / falo (sin prótesis)
☐ Próstata (probable que lo tenga/haya tenido si se le asignó sexo masculino al nacer)
☐ Testículos

Esta sección de la encuesta está destinada a darnos una idea de su salud física.

¿Alguna vez ha fumado cigarrillos, incluso una o dos bocanadas? ◯ Sí ◯ No

¿Ha fumado al menos 100 cigarrillos en TODA SU VIDA? ◯ Sí ◯ No

En el último MES, ¿ha consumido tabaco o productos de nicotina además de los cigarrillos?

☐ Blunt (con otra sustancia)
☐ Blunt (sin ninguna otra sustancia)
☐ Bidi
☐ Mascar tabaco ("masticar")
☐ Otros puros con Tabaco en su interior (p.Ej. cigarillos, puritos, bidis)
☐ Otros puros con otra sustancia (p.Ej. cigarillos, puritos, bidis)
☐ Dip
☐ Cigarrillo electrónico o dispositivo de vapeo con nicotina
☐ Cigarrillo electrónico o dispositivo vape sin nicotina
☐ Productos sustitutivos de la nicotina
☐ Snuff
☐ Snus
☐ Otro producto que contenga tabaco o nicotina (por favor especifique)
☐ Nunca he usado ningún producto de tabaco que no sean cigarrillos
☐ Nunca he usado ningún producto de tabaco o nicotina

Otro producto que contenga tabaco o nicotina (por favor especifique) ______________________

¿Con qué frecuencia tomó una bebida que contenía alcohol en el ÚLTIMO AÑO?

◯ Nunca
◯ Mensual o menos
◯ 2-4 veces al mes
◯ 2-3 veces por semana
◯ 4 o más veces a la semana

¿Cuántas bebidas que contenían alcohol tomó en un día típico cuando bebía en el AÑO PASADO?

◯ 1 o 2
◯ 3 o 4
◯ 5 o 6
◯ 7-9
◯ 10 o más

¿Cuánto tiempo ha pasado desde que bebió 5 o más bebidas que contenían alcohol en una ocasión?

◯ En los últimos 30 días
◯ Hace más de 30 días pero dentro de los últimos 12 meses
◯ Hace más de 12 meses
◯ Nunca tomé 5 o más tragos en una ocasión

Encuesta 25% Completa

¿Un proveedor de atención médica le ha diagnosticado cáncer? ◯ Sí ◯ No

¿Con qué tipo (s) de cáncer le han diagnosticado? (Marque todo lo que corresponda.)

- ☐ Anal
- ☐ Pecho
- ☐ Cuello uterino
- ☐ Colón
- ☐ Riñón
- ☐ Pulmón
- ☐ Leucemia / Linfoma
- ☐ Ovario
- ☐ Páncreas
- ☐ Próstata
- ☐ Piel (melanoma)
- ☐ Piel (no-melanoma)
- ☐ Útero
- ☐ Otro (por favor especifique)

Otro (por favor especifique) __________________________________

Ha indicado que actualmente tiene una vaginal /abertura genital frontal. Para personalizar el resto de este cuestionario, seleccione el término que le gustaría que usemos para describir su vagina / abertura genital frontal.

Porfavor use el término

- ◯ vagina
- ◯ abertura genital frontal

Esta sección pregunta sobre la detección del cáncer.
Por favor, haga todo lo posible para responder a todas las preguntas, pero puede omitir las preguntas que le resulten demasiado incómodas para responder. Completar todo el cuestionario significa que tenemos más poder para promover la salud LGBTQ +. ¡Gracias por hacer una diferencia!

¿Alguna vez te has hecho una prueba de Papanicolaou? (Una prueba de Papanicolaou es una prueba de rutina en la que un médico coloca un instrumento dentro de la vagina, examina el cuello uterino y extrae algunas células del cuello uterino con una pequeña varilla o cepillo para buscar células anormales o cancerosas).

◯ Sí
◯ No
◯ No lo sé

¿En los últimos 3 años, has hecho una prueba de Papanicolaou? (Una prueba de Papanicolaou es una prueba de rutina en la que un médico coloca un instrumento dentro de la vagina, examina el cuello uterino y extrae algunas células del cuello uterino con una pequeña varilla o cepillo para buscar células anormales o cancerosas).

◯ Sí
◯ No
◯ No lo sé

¿Cuánto tiempo ha pasado desde su última prueba de Papanicolaou?

◯ Hace un año o menos
◯ Hace más de 1 año pero no más de 2 años
◯ Hace más de 2 años pero no más de 3 años
◯ Hace más de 3 años pero no más de 5 años
◯ Hace más de 5 años
◯ No lo sé

¿Cuál es la razón más importante por la que NUNCA se ha hecho una prueba de Papanicolaou?

◯ No tengo un motivo o nunca lo pensé
◯ No sabía que necesitaba este tipo de prueba
◯ No sabía que existía esta prueba
◯ Mi proveedor de atención médica me dijo que no lo necesitaba
◯ No he tenido ningún problema
◯ Lo pospuse o no lo logré
◯ Demasiado caro
◯ No tengo seguro
◯ Fue demasiado doloroso, desagradable o vergonzoso
◯ No tengo cuello uterino o me han hecho una histerectomía
◯ No tengo proveedor (10)
◯ Tenía una vacuna contra el VPH
◯ Nadie que yo conozca ha tenido uno
◯ No lo sé
◯ Otro (por favor especifique)

Otro (por favor especifique) __________________________________

¿Cuál es la razón más importante por la que NO se ha hecho una prueba de Papanicolaou en los últimos 3 años?

- ◯ No tengo un motivo o nunca lo pensé
- ◯ No sabía que necesitaba este tipo de prueba
- ◯ No sabía que existía esta prueba
- ◯ Mi proveedor de atención médica me dijo que no lo necesitaba
- ◯ No he tenido ningún problema
- ◯ Lo pospuse o no lo logré
- ◯ Demasiado caro
- ◯ No tengo seguro
- ◯ Fue demasiado doloroso, desagradable o vergonzoso
- ◯ No tengo cuello uterino o me han hecho una histerectomía
- ◯ No tengo proveedor (10)
- ◯ Tenía una vacuna contra el VPH
- ◯ Nadie que yo conozca ha tenido uno
- ◯ No lo sé
- ◯ Otro (por favor especifique)

Otro (por favor especifique) ______________________________

¿Alguna vez se ha hecho una prueba de Papanicolaou donde los resultados NO fueron normales?

- ◯ Sí
- ◯ No
- ◯ No lo sé

VPH - Virus de Papiloma Humano

A veces se agrega una prueba de VPH a la prueba de Papanicolaou para la detección del cáncer de cuello uterino. ¿Alguna vez se ha hecho una prueba de VPH junto con su prueba de Papanicolaou cervical?

◯ Sí
◯ No
◯ No lo sé

¿Se hizo una prueba de VPH con una prueba de Papanicolaou en los ÚLTIMOS 5 AÑOS?

◯ Sí
◯ No
◯ No lo sé

¿Se hizo una prueba de VPH sin una prueba de Papanicolaou en los ÚLTIMOS 5 AÑOS?

◯ Sí
◯ No
◯ No lo sé

¿Alguna vez se ha realizado una prueba del VPH en la que los resultados fueron positivos (lo que significa que fue positivo para el virus del VPH)?

◯ Sí
◯ No
◯ No lo sé

¿Alguna vez te has hecho una mamografía? Una mamografía es cuando el tejido mamario / torácico se aprieta entre dos superficies firmes para obtener radiografías / imágenes del tejido mamario

◯ Sí
◯ No
◯ No lo sé

¿Cuál es la razón más importante por la que NUNCA se ha realizado una mamografía?

◯ No tengo un motivo o nunca lo pensé
◯ No sabía que necesitaba este tipo de prueba
◯ No sabía que existía esta prueba
◯ Mi proveedor de atención médica me dijo que no lo necesitaba
◯ No he tenido ningún problema
◯ Lo pospuse o no lo logré
◯ Demasiado caro
◯ No tengo seguro
◯ Fue demasiado doloroso, desagradable o vergonzoso
◯ No tengo cuello uterino o me han hecho una histerectomía
◯ No tengo proveedor
◯ Nadie que yo conozca ha tenido uno
◯ No lo sé
◯ Otro (por favor especifique)

Otro (por favor especifique)

____

En los ÚLTIMOS 2 AÑOS, ¿se ha realizado una mamografía? Una mamografía es cuando el tejido mamario / torácico se aprieta entre dos superficies firmes para obtener radiografías / imágenes del tejido mamario

◯ Sí
◯ No
◯ No lo sé

¿Se ha realizado una mamografía en los ÚLTIMOS 2 AÑOS en los que los resultados NO fueron normales?

◯ Sí
◯ No
◯ No lo sé

¿Cuánto tiempo ha pasado desde su última mamografía?

◯ Hace un año o menos
◯ Hace más de 1 año pero no más de 2 años
◯ Hace más de 2 años pero no más de 3 años
◯ Hace más de 3 años pero no más de 5 años
◯ Hace más de 5 años
◯ No lo sé

¿Cuál es la razón más importante por la que NO se ha realizado una mamografía en los ÚLTIMOS 2 AÑOS?

◯ No tengo un motivo o nunca lo pensé
◯ No sabía que necesitaba este tipo de prueba
◯ No sabía que existía esta prueba
◯ Mi proveedor de atención médica me dijo que no lo necesitaba
◯ No he tenido ningún problema
◯ Lo pospuse o no lo logré
◯ Demasiado caro
◯ No tengo seguro
◯ Fue demasiado doloroso, desagradable o vergonzoso
◯ No tengo cuello uterino o me han hecho una histerectomía
◯ No tengo proveedor
◯ Nadie que yo conozca ha tenido uno
◯ No lo sé
◯ Otro (por favor especifique)

Otro (por favor especifique)

____

¿Alguna vez se hizo una mamografía en la que los resultados NO fueron normales?

◯ Sí
◯ No
◯ No lo sé

¿Alguna vez te has hecho una prueba de PSA? Una prueba de PSA es un análisis de sangre para detectar el cáncer de próstata. También se llama prueba de antígeno prostático específico

◯ Sí
◯ No
◯ No lo sé

¿Cuál es la razón más importante por la que NUNCA se ha realizado una prueba de PSA?

◯ No tengo un motivo o nunca lo pensé
◯ La prueba no es recomendada para mi
◯ No sabía que necesitaba este tipo de prueba
◯ No sabía que existía esta prueba
◯ Mi proveedor de atención médica me dijo que no lo necesitaba
◯ No he tenido ningún problema
◯ Lo pospuse o no lo logré
◯ Demasiado caro
◯ No tengo seguro
◯ Fue demasiado doloroso, desagradable o vergonzoso
◯ No tengo proveedor
◯ Nadie que yo conozca ha tenido uno
◯ No lo sé
◯ Otro (por favor especifique)

Otro (por favor especifique)

____

¿Quién sugirió primero la prueba de PSA?

◯ Yo
◯ Mi proveedor de atención médica
◯ Alguien más
◯ No lo sé

¿Algún médico o proveedor de atención médica le habló ALGUNA VEZ sobre las ventajas de la prueba de PSA?

◯ Sí
◯ No
◯ No lo sé

En los ÚLTIMOS 12 MESES, ¿le hicieron una prueba de PSA?

◯ Sí
◯ No
◯ No lo sé

¿Ha tenido una prueba de PSA en los ÚLTIMOS 12 MESES donde los resultados NO fueron normales?

◯ Sí
◯ No
◯ No lo sé

Encuesta 50% Completa
Las pruebas de cáncer de colon o recto incluyen análisis de sangre en las heces, colonoscopia y sigmoidoscopia. Una prueba de sangre en las heces o una prueba de sangre oculta, también conocida como prueba inmunoquímica fecal (FIT), determina si tiene sangre en las heces. Estas pruebas se pueden realizar en casa con un kit. Utiliza un palillo o un cepillo para obtener una pequeña cantidad de materia fecal en casa y enviarla al médico o al laboratorio. Una sigmoidoscopia y una colonoscopia son exámenes en los que se inserta un tubo en el recto para observar el colon en busca de signos de cáncer u otros problemas de salud. Antes de una sigmoidoscopia o colonoscopia, se le pide que tome un medicamento que cause diarrea. Para una sigmoidoscopia, el médico u otro proveedor de atención médica revisa solo una parte del colon y usted está completamente despierto. Para una colonoscopia, el médico u otro proveedor de atención médica revisa todo el colon y le administran un medicamento a través de una aguja en el brazo para adormecerlo y le dicen que alguien lo lleve a su casa.

¿Alguna vez se ha realizado alguna de estas pruebas para detectar cáncer de colon o recto? (Marque todo lo que corresponda.)

☐ Ninguno de estos
☐ Prueba de heces de sangre (prueba FIT)
☐ Sigmoidoscopia
☐ Colonoscopia

¿Cuál es la razón más importante por la que NUNCA se ha realizado ninguna de estas pruebas para detectar cáncer de colon o recto?

◯ No tengo un motivo o nunca lo pensé
◯ No sabía que necesitaba este tipo de prueba
◯ No sabía que existía esta prueba
◯ Mi proveedor de atención médica me dijo que no lo necesitaba
◯ No he tenido ningún problema
◯ Lo pospuse o no lo logré
◯ Demasiado caro
◯ No tengo seguro
◯ Fue demasiado doloroso, desagradable o vergonzoso
◯ No tengo proveedor
◯ Tengo menos de 50 años
◯ Nadie que yo conozca ha tenido uno
◯ No lo sé
◯ Otro (por favor especifique)

Otro (por favor especifique) __________________________________

¿Cuánto tiempo ha pasado desde su último análisis de heces de sangre?

◯ Hace un año o menos
◯ Hace más de 1 año pero no más de 2 años
◯ Hace más de 2 años pero no más de 3 años
◯ Hace más de 3 años pero no más de 5 años
◯ Hace más de 5 años pero no más de 10 años
◯ Hace más de 10 años
◯ No lo sé

¿Cuál es la razón más importante por la que NO se ha hecho una prueba de sangre en las heces (prueba FIT) en el ÚLTIMO 1 AÑO?

◯ No tengo un motivo o nunca lo pensé
◯ No sabía que necesitaba este tipo de prueba
◯ No sabía que existía esta prueba
◯ Mi proveedor de atención médica me dijo que no lo necesitaba
◯ No he tenido ningún problema
◯ Lo pospuse o no lo logré
◯ Demasiado caro
◯ No tengo seguro
◯ Fue demasiado doloroso, desagradable o vergonzoso
◯ No tengo proveedor
◯ Tengo menos de 50 años
◯ Tengo más de 75 años
◯ Nadie que yo conozca ha tenido uno
◯ No lo sé
◯ Otro (por favor especifique)

Otro (por favor especifique)

____

¿Alguna vez se ha realizado una prueba de sangre en las heces (FIT) donde los resultados NO fueron normales?

◯ Sí
◯ No
◯ No lo sé

¿Cuánto tiempo ha pasado desde su última sigmoidoscopia?

◯ Hace un año o menos
◯ Hace más de 1 año pero no más de 2 años
◯ Hace más de 2 años pero no más de 3 años
◯ Hace más de 3 años pero no más de 5 años
◯ Hace más de 5 años pero no más de 10 años
◯ Hace más de 10 años
◯ No lo sé

¿Cuál es la razón más importante por la que NO se ha realizado una sigmoidoscopia en los ÚLTIMOS 10 AÑOS?

◯ No tengo un motivo o nunca lo pensé
◯ No sabía que necesitaba este tipo de prueba
◯ No sabía que existía esta prueba
◯ Mi proveedor de atención médica me dijo que no lo necesitaba
◯ No he tenido ningún problema
◯ Lo pospuse o no lo logré
◯ Demasiado caro
◯ No tengo seguro
◯ Fue demasiado doloroso, desagradable o vergonzoso
◯ No tengo proveedor
◯ Nadie que yo conozca ha tenido uno
◯ No lo sé
◯ Otro (por favor especifique)

Otro (por favor especifique)

____

¿Alguna vez se ha sometido a una sigmoidoscopia donde los resultados NO fueron normales?

◯ Sí
◯ No
◯ No lo sé

¿Cuánto tiempo ha pasado desde su última colonoscopia?

◯ Hace un año o menos
◯ Hace más de 1 año pero no más de 2 años
◯ Hace más de 2 años pero no más de 3 años
◯ Hace más de 3 años pero no más de 5 años
◯ Hace más de 5 años pero no más de 10 años
◯ Hace más de 10 años
◯ No lo sé

Cuál es la razón más importante por la que NO se ha realizado una prueba de colonoscopia en los ÚLTIMOS 10 AÑOS?

◯ No tengo un motivo o nunca lo pensé
◯ No sabía que necesitaba este tipo de prueba
◯ No sabía que existía esta prueba
◯ Mi proveedor de atención médica me dijo que no lo necesitaba
◯ No he tenido ningún problema
◯ Lo pospuse o no lo logré
◯ Demasiado caro
◯ No tengo seguro
◯ Fue demasiado doloroso, desagradable o vergonzoso
◯ No tengo proveedor
◯ Nadie que yo conozca ha tenido uno
◯ No lo sé
◯ Otro (por favor especifique)

Otro (por favor especifique)

____

¿Alguna vez se ha realizado una colonoscopia en la que los resultados NO fueron normales?

◯ Sí
◯ No
◯ No lo sé

¿Alguna vez se ha sometido a alguna de las siguientes pruebas como evaluación de cáncer de recto o anal?(Marque todo lo que corresponda.)

☐ Examen rectal anal digital (un examen en el que un médico o proveedor de atención médica inserta su dedo en su ano (trasero)
☐ Prueba anal de VPH (una prueba de rutina con un hisopo que prueba el virus del papiloma humano, VPH)
☐ Prueba de Papanicolaou anal (una prueba de rutina en la que un proveedor de atención médica extrae algunas células del ano (trasero) con un hisopo para buscar células anormales o cancerosas)
☐ Anoscopia de alta resolución (HRA) (un examen con un microscopio del recto y el ano / trasero)
☐ No lo sé
☐ Ninguno de estos

¿Alguna vez se ha realizado una prueba anal del VPH en la que los resultados NO fueron normales?

◯ Sí
◯ No
◯ No lo sé

¿Alguna vez se hizo una prueba de Papanicolaou anal donde los resultados NO fueron normales?(ANOREC_SCREEN_PAP_L)

◯ Sí
◯ No
◯ No lo sé

¿Alguna vez se ha sometido a una tomografía computarizada de dosis baja (TC o TAC) para detectar cáncer de pulmón?

◯ Sí
◯ No
◯ No lo sé

¿Alguna vez se ha realizado una tomografía computarizada donde los resultados NO fueron normales?

◯ Sí
◯ No
◯ No lo sé

¿Cuánto tiempo ha pasado desde su última tomografía computarizada?

◯ Hace un año o menos
◯ Hace más de 1 año pero no más de 2 años
◯ Hace más de 2 años pero no más de 3 años
◯ Hace más de 3 años pero no más de 5 años
◯ Hace más de 5 años
◯ No lo sé

¿Cuál es la razón más importante por la que NUNCA se ha realizado una prueba de TC?

◯ No tengo un motivo o nunca lo pensé
◯ No sabía que necesitaba este tipo de prueba
◯ Mi proveedor de atención médica me dijo que no lo necesitaba
◯ No he tenido ningún problema
◯ Lo pospuse o no lo logré
◯ Demasiado caro
◯ No tengo seguro
◯ No tengo proveedor
◯ No lo sé
◯ Otro (por favor especifique)

Otro (por favor especifique) __________________________________

Encuesta 75% Completa

¿Alguna vez recibió una inyección o vacuna contra el VPH? VPH significa virus del papiloma humano. Las vacunas a veces se llaman CERVARIX® o GARDASIL®. La vacuna contra el VPH se administra en una serie de tres dosis de forma rutinaria a personas de entre 9 y 45 años. Fue lanzado en 2006.

◯ Sí
◯ No
◯ No lo sé

¿Cuál es la razón más importante por la que NUNCA recibió la vacuna contra el VPH?

◯ No tengo un motivo o nunca lo pensé
◯ No sabía que necesitaba esta vacuna
◯ No sabía que existía esta vacuna
◯ El médico u otro proveedor de atención médica se negó a dármelo cuando lo pedí
◯ Mi proveedor de atención médica me dijo que no lo necesitaba
◯ Lo pospuse o no lo logré
◯ Demasiado caro
◯ No tengo seguro
◯ Fue demasiado doloroso, desagradable o vergonzoso
◯ No tengo cuello uterino o me han hecho una histerectomía
◯ No tengo proveedor
◯ Nadie que yo conozca ha tenido uno
◯ No lo sé
◯ Otro (por favor especifique)

Otro (por favor especifique)

____

¿Cuántas vacunas contra el VPH se puso?

◯ Una
◯ Dos
◯ Tres
◯ No lo sé

Esta última sección de la encuesta está destinada para darnos una idea de su acceso a atención médica.

Muchas de estas preguntas son preguntas estándar que se formulan de forma rutinaria en las encuestas nacionales de salud. Sus respuestas honestas nos ayudarán mientras estudiamos la salud LGBTQ + para mejorar la salud y el bienestar de nuestras comunidades en Nuevo México.

¿Está cubierto actualmente por algún seguro médico o plan de cobertura médica?

◯ Sí
◯ No
◯ No lo sé

¿Está actualmente cubiertx por alguno de los siguientes tipos de seguro médico o planes de cobertura médica? (Si tiene más de un plan de seguro / cobertura, seleccione su plan de seguro / cobertura principal).

◯ Seguro a través de mi empleador o sindicato actual o anterior
◯ Seguro a través del empleador o sindicato actual o anterior de otra persona
◯ Seguro comprado a través de CuidadoDeSalud.gov u otro mercado de seguros de salud (a veces llamado "Obamacare" o la "Ley de Cuidado de Salud Asequible")
◯ Seguro adquirido directamente de una compañía de seguros
◯ Médicare (para personas mayores de 65 años o personas con ciertas discapacidades)
◯ Medicaid (plan de asistencia del gobierno para personas con bajos ingresos o una discapacidad)
◯ TRICARE u otra atención médica militar
◯ Veteran's Affair (VA)
◯ Servicio de salud indígena
◯ Otro (por favor especifique)

Otro (por favor especifique) __________________________________

¿Estuvo sin seguro en algún momento durante los últimos 12 meses?

◯ Sí
◯ No
◯ No lo sé

¿Hay algún lugar al que VAYA GENERALMENTE cuando necesite atención de rutina o preventiva, como un examen físico o chequeo?

◯ Sí
◯ No
◯ No lo sé

Durante los últimos 12 meses, ¿tuvo problemas para encontrar un médico general o un proveedor de atención médica que lo atendiera?

◯ Sí
◯ No
◯ No he intentado ver a un médico o proveedor de atención médica en los últimos 12 meses.
◯ No lo sé

Si ha tenido problemas para encontrar un médico generalista o un proveedor de atención médica en los últimos 12 meses, ¿cuál es el motivo?

◯ El proveedor más cercano estaba demasiado lejos
◯ No pensé que lo necesitaba
◯ Me preocupa la discriminación
◯ Es demasiado caro
◯ No tengo transporte confiable
◯ No me fío del sistema sanitario
◯ Tengo otras prioridades
◯ No tengo seguro médico
◯ No confío en los proveedores locales
◯ Otro (por favor especifique)

Otro (por favor especifique) ________________________________

Un proveedor de atención primaria es un proveedor de atención médica que se ocupa de su salud general en general y puede coordinar su atención con otros especialistas médicos. ¿Tiene un proveedor de atención primaria?

◯ Sí
◯ No
◯ No lo sé

¿Ha visto a su proveedor de atención primaria en los ÚLTIMOS 12 MESES?

◯ Sí
◯ No
◯ No lo sé

En los ÚLTIMOS 12 MESES, ¿se retrasó en recibir atención médica, pruebas o tratamientos que usted o un proveedor de atención médica consideraron necesarios?

◯ Sí
◯ No
◯ No aplica

En los ÚLTIMOS 12 MESES, ¿no pudo obtener la atención médica, las pruebas o los tratamientos que usted o un proveedor de atención médica consideraron necesarios?

◯ Sí
◯ No
◯ No aplica

En los ÚLTIMOS 12 MESES, ¿le han negado o le han brindado atención médica de baja calidad?

◯ Sí
◯ No
◯ No aplica

¿Cree que fue discriminadx en un entorno médico en los ÚLTIMOS 12 meses debido a su... (Marque todo lo que corresponda)

☐ Estado de capacidad / discapacidad
☐ Edad
☐ Tamaño, peso o forma corporal
☐ Expresión de género
☐ Identidad de género
☐ Raza y / o etnia
☐ Orientación sexual
☐ Algo más (por favor específico)
☐ Ninguno de los anteriores

Something else (please specify) __________________________________

¿Hay algo más que le gustaría compartir con nosotros sobre su salud o bienestar?

Encuesta 90% Completa Por favor lea a continuación e indique su interés.

Esta encuesta es parte de un proyecto de investigación más amplio que intenta comprender cuál es la mejor manera de promover la detección del cáncer de cuello uterino entre lxs mujeres lesbianas, bisexuales, queer, cisgénero, hombres transgénero, e individuos con cuello uterino que no se conforman a un género. Esperamos hablar con las personas de la comunidad para mejor comprender algunas de las barreras para obtener atención y los problemas que podrían impedir que las personas de estos grupos accedan a la detección del cáncer de cuello uterino.

Esta parte del estudio consistirá en participar en un grupo de discusión con algunos otros participantes y un entrevistador. Participación en este grupo de enfoque será anónima y requerirá que responda las preguntas publicadas por el entrevistador e interactúe con otros participantes.

¿Estaría dispuesto a hablar con nosotros sobre sus experiencias en la búsqueda de servicios de detección del cáncer del cuello utero?

○ Sí ○ No

Proporcione un correo electrónico o un número de teléfono al que podamos comunicarnos con usted. (No compartiremos esta información con nadie fuera del equipo del estudio y esta información no estará vinculada a las respuestas que proporcionó anteriormente)

¡Gracias por su participación!

¿Cómo le gustaría que le enviemos esta tarjeta de mercancía?

◯ Código de tarjeta de mercancía en línea ◯ Correo postal

Proporcione un correo electrónico donde podamos enviar su tarjeta de mercancía. (No compartiremos esta información con nadie fuera del equipo del estudio y esta información no estará vinculada a las respuestas que proporcionó anteriormente)

____

Proporcione la dirección postal a la que podamos enviar su tarjeta de mercancía. (No compartiremos esta información con nadie fuera del equipo del estudio y esta información no estará vinculada a las respuestas que proporcionó anteriormente)

Dirección postal / apartado postal __________________________________

Ciudad __________________________________

Estado __________________________________

Codigo postal __________________________________

Ha terminado con la encuesta.

Estamos comprometidos a comunicar los hallazgos del estudio a nuestra comunidad.

Como se mencionó anteriormente, su respuesta a la encuesta es anónima, lo que significa que no aparecerá información identificable individual en documentos de investigación, presentaciones o publicaciones.

Los datos individuales recopilados a través de esta encuesta se agregarán y se utilizarán para informes de investigación.

Si está interesado en obtener más información sobre nuestro estudio y los resultados del estudio, responda las siguientes preguntas.

¿Le gustaría saber sobre los hallazgos del estudio? ○ Sí ○ No

¿Cómo le gustaría que le enviemos comunicaciones futuras? ○ Correo electrónico ○ Correo postal

Proporcione un correo electrónico donde podamos enviarle comunicaciones futuras. (No compartiremos esta información con nadie fuera del equipo del estudio y esta información no estará vinculada a las respuestas que proporcionó anteriormente)

____

Proporcione la dirección postal a la que podamos enviarle comunicaciones en el futuro. (No compartiremos esta información con nadie fuera del equipo del estudio y esta información no estará vinculada a las respuestas que proporcionó anteriormente)

Dirección postal / apartado postal __________________________________

Ciudad __________________________________

Estado __________________________________

Codigo postal __________________________________

Encuesta 100% Completa

¡Gracias por hacer una diferencia!

en el futuro, también queremos conectar a nuestros participantes con algunos recursos que pueden ser útiles para ellos ahora. A continuación, encontrará una lista de sitios web, organizaciones y líneas directas que pueden ser útiles para promover la salud, la seguridad y el bienestar de las personas LGBTQ.

• Encuentre pruebas de VIH gratuitas en su área a través del programa GetTested de los Centros para el Control de Enfermedades: https://gettested.cdc.gov/

• Encuentre un médico amigable con LGBTQ + a través de GLMA: Health Professionals Advancing LGBT Equality: https://glmaimpak.networkats.com/members_online_new/members/dir_provider.asp

Recursos del área de Albuquerque:
• Centro de Recursos Transgénero de Nuevo México: https://tgrcnm.org/; (505) 200-9086
• Hable con alguien las 24 horas del día, los 7 días de la semana si está en crisis o está pensando en suicidarse: Línea Nacional de Prevención del Suicidio: 1-800-273-8255

• Centro de crisis de UNM AGORA: http://www.agoracares.org/; (505) 277-3013
• Hable con alguien las 24 horas del día, los 7 días de la semana si necesita apoyo relacionado con ser un sobreviviente de agresión sexual: Línea directa nacional de agresión sexual: 1-800-656-4673

• Colaborativo de Albuquerque SANE: https://abqsane.org/; (505) 884-SANE

Gracias nuevamente por completar la encuesta.

Apreciamos profundamente por su tiempo, su interés en este estudio y su inversión en la investigación que ayudará a nuestras comunidades a entender cómo la experiencia de ser LGBTQ + está relacionada con todos los aspectos de la salud y la vida.

Por favor seleccione "SUBMIT"

Por favor use esté espacio para comentarios

CLICK "SUBMIT" TO COMPLETE SURVEY
