# Supplementary material for: Reaching the “Hard-to-Reach” Sexual and Gender Diverse Communities for Population-Based Research in Cancer Prevention and Control: Methods for Online Survey Data Collection and Management
Source: Front Oncol. 2022 Jun 8;12:841951. doi: 10.3389/fonc.2022.841951 (PMC9213655; doi:10.3389/fonc.2022.841951)
Supplement: Supplementary Appendix 2B — Cancer prevention practices survey in Spanish. [file DataSheet_4.pdf]

CACTII-SGM Encuesta de Eligibilidad

¡Sí, quiero mejorar los servicios de detección del cáncer para la comunidad LGBTQI en Nuevo México!

Status

◯ Interested/ Elegible
◯ Survey sent - Complete
◯ Archive

¡Bienvenidos al estudio CACTII - SGM!

Gracias por su interés en participar en este estudio importante.

El estudio CACTII-SGM tiene como objetivo comprender los comportamientos de detección del cáncer entre las comunidades de minorías sexuales y de género en Nuevo México, que incluye a personas que se identifican como lesbianas, gays, bisexuales, transgénero, queer o de dos espíritus. Los resultados de esta encuesta nos permitirán comprender cuál es la mejor manera de promover la detección del cáncer en esta población.
La Oficina de Protección de la Investigación Humana de la Universidad de Nuevo México aprobó este estudio. (Numero de Estudio HRRC 20-393)

---

1. ¿Cómo se enteró de este estudio?
(Marque todo lo que corresponda)

☐ Vi una publicación en las redes sociales (Facebook o Google)
☐ Recibí un correo electrónico sobre el estudio
☐ Recibí un volante por correo
☐ Un familiar / amigo / vecino / colega me habló de este estudio
☐ Otro (Especifique)

Otro (por favor especifique) ______________________________

---

2. ¿Nació entre los años 1940 y 1999?

◯ Sí ◯ No

---

3. ¿Te identificas como lesbiana, gay, bisexual, transgénero, queer o de dos espíritus?[

◯ Sí ◯ No

---

4. ¿Eres residente de Nuevo México?

◯ Sí ◯ No

¡Eres elegible para este estudio! Si aún está interesado en participar, seleccione una opción a continuación.

El siguiente paso de este estudio es responder una encuesta que contiene preguntas sobre su cuerpo y comportamientos de salud relacionados con la detección del cáncer. Esperamos crear una comprensión integral de la salud de nuestras comunidades LGBTQ + en lo que respecta a la prevención del cáncer.

Estimamos que esta encuesta tomará entre 15 y 20 minutos en completarse. .

Al completar la encuesta, será elegible para recibir una tarjeta de mercancía de $ 20 como compensación por su tiempo.

---

5. ¿Le gustaría participar en el estudio? ◯ Sí ◯ No

6. ¿Cuál es su idioma preferido para responder la encuesta?	◯ Ingles ◯ Español
7. ¿Cómo le gustaría recibir la encuesta?	◯ Correo electrónico ◯ Correo postal
8. Proporcione una dirección de correo electrónico para recibir la encuesta.	__________________________
9. Proporcione una dirección de correo postal para recibir la encuesta.	
Calle y número de casa o Apartado de correos:	__________________________
Ciudad	__________________________
Código postal:	__________________________

No eres elegible para este estudio
Si tiene alguna pregunta o sugerencia para nosotros. Puede comunicarse con el coordinador del estudio al [505-925-0619]
Les agradecemos de antemano por promover este importante trabajo que ayudará a nuestras comunidades a comprender cómo la experiencia de ser LGBTQ + se relaciona con la prevención del cáncer.
A continuación, encontrará una lista de sitios web, organizaciones y líneas directas que pueden ser útiles para promover la salud, la seguridad y el bienestar de las personas LGBTQ.

• Encuentre pruebas de VIH gratuitas en su área a través del programa GetTested de los Centros para el Control de Enfermedades: https://gettested.cdc.gov/

• Encuentre un médico amigable con LGBTQ + a través de GLMA: Health Professionals Advancing LGBT Equality: https://glmaimpak.networkats.com/members_online_new/members/dir_provider.asp

Recursos del área de Albuquerque:

• Centro de Recursos Transgénero de Nuevo México: https://tgrcnm.org/; (505) 200-9086

• Hable con alguien las 24 horas del día, los 7 días de la semana si está en crisis o está pensando en suicidarse: Línea Nacional de Prevención del Suicidio: 1-800-273-8255

• Centro de crisis de UNM AGORA: : http://www.agoracares.org/; (505) 277-3013

• • Hable con alguien las 24 horas del día, los 7 días de la semana si necesita apoyo relacionado con ser un sobreviviente de agresión sexual: Línea directa nacional de agresión sexual: 1-800-656-4673

• • Colaborativo de Albuquerque SANE: https://abqsane.org/; (505) 884-SANE

SELECCIONE 'SUBMIT'
